# Supplementary material for: Converting inorganic sulfur into degradable thermoplastics and adhesives by copolymerization with cyclic disulfides
Source: Nat Commun. 2024 May 8;15:3855. doi: 10.1038/s41467-024-48097-4 (PMC11079033; doi:10.1038/s41467-024-48097-4)
Supplement: Supplementary file 1 — Supplementary Information [file 41467_2024_48097_MOESM1_ESM.pdf]

Supplementary Information

Converting inorganic sulfur into degradable thermoplastics and adhesives by copolymerization with cyclic disulfides

Yuanxin Deng[1], Zhengtie Huang[1], Ben L. Feringa[1,2]✉, He Tian[1], Qi Zhang[1]✉, Da-Hui Qu[1]✉

[1]Key Laboratory for Advanced Materials and Joint International Research Laboratory of Precision Chemistry and Molecular Engineering, Feringa Nobel Prize Scientist Joint Research Center, Institute of Fine Chemicals, Frontiers Science Center for Materiobiology and Dynamic Chemistry, School of Chemistry and Molecular Engineering, East China University of Science and Technology; Meilong Road 130, Shanghai 200237, China.

[2] Stratingh Institute for Chemistry, Faculty of Science and Engineering, University of Groningen; Nijenborgh 4, 9747 AG Groningen, The Netherlands.

✉Correspondence to: b.l.feringa@rug.nl; q.zhang@ecust.edu.cn; dahui_qu@ecust.edu.cn

1. General Information and Supplementary Methods

1.1 Materials

The key feedstock (±)-α-thioctic acid (TA) was used as received from Aladdin with a Reagent Grade (99%). N,N'-disuccinimidyl carbonate (NHS), triethylamine (>99%), diethylamine (>99%), n-butylamine (>99%), ammonium hydroxide solution (28%), hydrazine monohydrate (85%), 4-(N,N-dimethylamino)pyridine (DMAP), N-(3-dimethylamino-propyl)-N'-ethylcarbodiimide hydrochloride (EDCI), m-diisopryl benzene (DIB), sublimed sulfur (99.5%), hexamethylphosphoramide (98%), P-toluidine (99%), 5-hexen-1-ol (97%), TEMPO, chloranil (96%), and hydroquinone (99%) were obtained from commercial sources (Adamas®beta, TCI, Thermo Fisher, Aladdin, and HUSHI) and used as received without further purification. Solvents used for reaction and spectroscopy were AR or HPLC grade. Solvents for NMR spectroscopy were used as received from CIL.

1.2 Compound characterization

^{1}H and ^{13}C Solution State Nuclear Magnetic Resonance (NMR) Spectroscopy. ^{1}H NMR and ^{13}C NMR spectra were measured on a Bruker AV-400 (^{1}H: 400 MHz, ^{13}C: 100 MHz) spectrometer at room temperature. Chemical shift values (δ) are reported in parts per million (ppm) with the solvent resonance as the internal standard (DMSO: δ 2.50 for ^{1}H, δ 39.52 for ^{13}C, $CDCl_3$: δ 7.26 for ^{1}H, δ 77.16 for ^{13}C, CD_2Cl_2: δ 5.32 for ^{1}H, δ 53.84 for ^{13}C). The following abbreviations (and their corresponding combination) are used to indicate signal multiplicity: s (singlet), d (doublet), t (triplet), q (quartet), m (multiplet), and br (broad).

Electrospray Ionization Mass Spectrometry (ESI-MS). ESI Mass Spectra were recorded on a ThermoFisher instrument Q Exactive Plus (ESI+).

2. Experimental Method

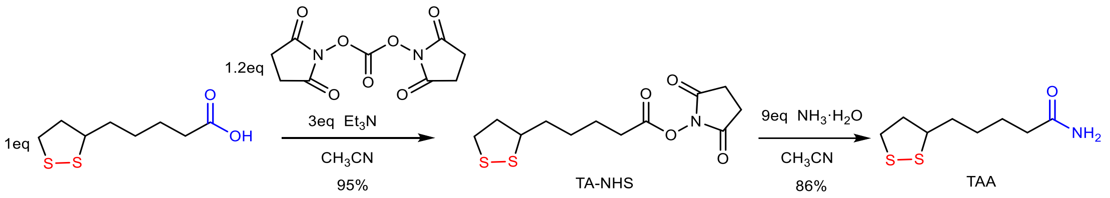

Preparation of TAA monomer: TAA was synthesized according to the previous literature.[1] (±)-α-thioctic acid (TA) (1.0 eq, 4.8 g, 23.0 mmol) was dissolved in CH_3CN (200 mL). Di-(N,N′-succinimidyl) carbonate (1.2 eq, 7.2 g, 28.0 mmol) and triethylamine (Et_3N, 3.0 eq, 10.0 mL) were added into the above solution and the mixture was stirred at room temperature for 2 h. The mixture was concentrated at 35 °C under reduced pressure and then added to an aqueous 5% $NaHCO_3$ solution to produce yellow precipitates (TA-NHS). The precipitates were filtered, washed by water, and then dissolved in CH_3CN (200 mL). 15 mL 25% ammonia solution (9.0 eq, 14.0 g, 0.2 mol) was dropwise added into the above solution and the mixture was stirred at room temperature for 5 h. The mixture was filtered and the filtrate was concentrated under reduced pressure and then recrystallized from CH_3CN twice at -25 °C to yield a yellow solid TAA monomer (3.4 g, 86%).

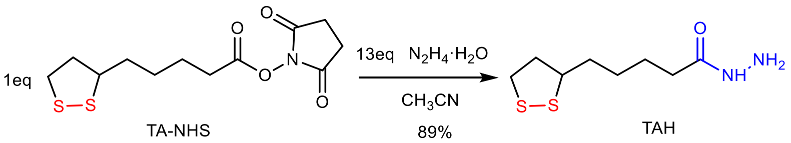

Preparation of TAH monomer: TAH was synthesized according to the previous literature.[1] TA-NHS (1.0 eq, 13.6 g, 45.0 mmol) was dissolved in CH_3CN (300 mL). 80% hydrazine monohydroxide (13.0 eq, 30 mL) was dropwise added into the solution and the mixture was stirred at room temperature for 3 h. The mixture was filtered and the filtrate was concentrated under reduced pressure. The concentrated solution was diluted with CH_2Cl_2 (200 mL) and washed by aqueous 5% $NaHCO_3$ solution three times. After drying over Na_2SO_4, the solvent was evaporated under reduced pressure to yield yellow solid TAH monomers (8.8 g, 89%).

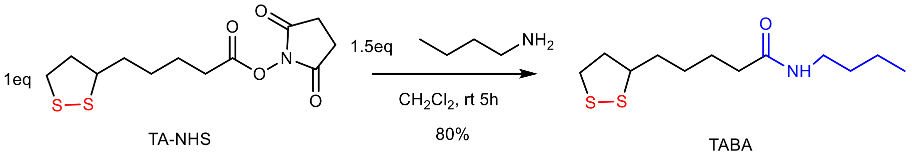

Preparation of TABA monomer: TA-NHS (1.0 eq, 6.0 g, 20.0 mmol) was dissolved in CH_2Cl_2 (160 mL). 3.0 mL n-butylamine (1.5 eq, 2.2 g, 30.0 mmol) was dropwise added into the above solution and the mixture was stirred at room temperature for 5 h. The solution was then washed by aqueous saturated NaCl solution three times. After dried over Na_2SO_4, the solvent was evaporated under reduced pressure to obtain a yellow solid TABA monomer (4.2 g, 80%). ^1H NMR (400 MHz, $CDCl_3$) δ 6.17 (s, 1H),

3.46 (m, 1H), 3.12 (m, 2H), 3.03 (m, 2H), 2.35 (m, 1H), 2.08 (t, 2H), 1.80 (m, 1H), 1.57 (m, 4H), 1.37 (m, 4H), 1.24 (m, 4H), 0.81 (t, 3H). ^{13}C NMR (600 MHz, $CDCl_3$) δ 172.76, 56.45, 40.24, 39.21, 38.48, 36.48, 34.64, 31.71, 28.91, 25.51, 20.09, 13.78. HRMS (ESI) (m/z): $[M + H]^+$ calcd for $C_{12}H_{23}NOS_2$ + H: 262.1294, found 262.1292.

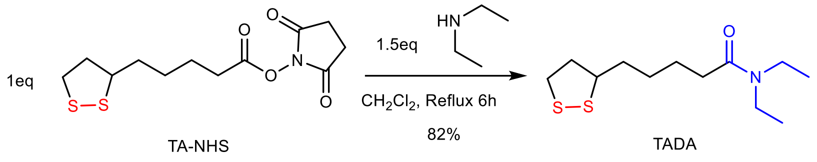

Preparation of TADA monomer: TA-NHS (1.0 eq, 6.0 g, 20.0 mmol) was dissolved in CH_2Cl_2 (160 mL). 3 mL diethylamine (1.5 eq, 2.2 g, 30.0 mmol) was dropwise added into the above solution at room temperature, and then the mixture was heated (37 °C) at reflux for 6h. After cooling, the solution was washed by aqueous saturated NaCl solution three times. After drying over Na_2SO_4, the solution was evaporated under reduced pressure to obtain yellow oil TADA monomer (4.3 g, 82%). ^{1}H NMR (400 MHz, $CDCl_3$) δ 3.50 (m, 1H), 3.27 (m, 2H), 3.21 (m, 2H), 3.06 (m, 2H), 2.38 (m, 1H), 2.22 (t, 2H), 1.83 (m, 1H), 1.60 (m, 4H), 1.39 (m, 2H), 1.08 (t, 3H), 1.01 (t, 3H). ^{13}C NMR (600 MHz, $CDCl_3$) δ 171.75, 56.43, 40.18, 40.03, 38.46, 34.75, 32.78, 29.08, 25.08, 14.38, 13.09. HRMS (ESI) (m/z): $[M + H]^+$ calcd for $C_{12}H_{23}NOS_2$ + H: 262.1294, found 262.1292.

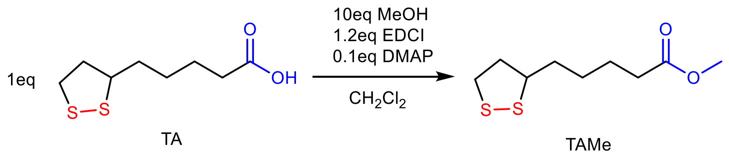

Preparation of TAMe monomer: TAMe was synthesized according to the previous literature.[2] (±)-α-Thioctic acid (5.0 g, 24.2 mmol, 1.0 eq) was dissolved in 300 mL CH_2Cl_2 while stirring under ice bath. 4-(N,N-dimethylamino)pyridine (DMAP, 0.3 g, 2.4 mmol, 0.1 eq) and N-(3-dimethylamino-propyl)-N'-ethylcarbodiimide hydrochloride (EDCI, 5.6 g, 29.0 mmol, 1.2 eq) were slowly added into the solution and followed by dropwise addition of methanol (7.8 g, 242.5 mmol, 10.0 eq). The mixture was stirred overnight. The solution was evaporated under reduced pressure and the yellow wax was dissolved in ethyl acetate and washed by 1 M HCl aqueous solution, deionized water, saturated aqueous $NaHCO_3$ solution, and saturated aqueous NaCl solution three times, respectively. The solution was evaporated under reduced pressure and the mixture was purified by column chromatography to obtain yellow oil TAMe (4.9 g, 91%).

Polymerization procedure:

In a typical procedure, 2 g (7.8 mmol) of S_8 monomer was added into a Teflon vial, which was then heated by a metal heating block at a constant temperature of 120 °C. Yellow viscous liquid S_8 was obtained under magnetic stirring. In the case of

polymerization, a given amount of comonomer was added to the molten S_8 liquid and dissolved by vigorous stirring. Next, the reaction mixture was stirred at 120 °C for 2 hours to obtain homogeneity, and the resulting liquid was quickly transferred into a Teflon mold and cooled to room temperature to form free-standing polymer samples.

Preparation of poly(S_8/TAA): The polymerization was carried out by following the general method mentioned above with various weight ratios of TAA. The weight ratio of S_8 to TAA is 3 to 1, 2 to 1, 1 to 1, 1 to 2, 1 to 3, 1 to 5, 1 to 10, 1 to 20, 1 to 30, respectively. When the weight ratio of S_8 to TAA is higher than 1 to 1, partial S_8 precipitated after cooling down to room temperature resulting in heterogeneous copolymers. When the weight ratio of S_8 to TAA is 1 to 2, 1 to 3, and 1 to 5, the resulting copolymers are red translucent thermoplastics. When the weight ratio of S_8 to TAA is 1 to 10, 1 to 20, and 1 to 50, the resulting copolymers are opaque and brittle solid.

Preparation of poly(S_8/TABA): The polymerization was carried out by following the general method mentioned above with various weight ratios of TABA. The weight ratio of S_8 to TABA is 2 to 1, 1 to 1, 1 to 2, 1 to 3, 1 to 5, respectively. When the weight ratio of S_8 to TABA is 2 to 1, 1 to 1, and 1 to 2, the resulting copolymers are light yellow and opaque viscous elastics. When the weight ratio of S_8 to TABA is 1 to 3 and 1 to 5, the resulting copolymers are red translucent viscous elastics.

Preparation of poly(S_8/TADA): The polymerization was carried out by following the general method mentioned above with various weight ratios of TADA. The weight ratio of S_8 to TADA is 2 to 1, 1 to 1, 1 to 2, 1 to 3, and 1 to 5, respectively. When the weight ratio of S_8 to TADA is 2 to 1 and 1 to 1, the resulting copolymers are light yellow and opaque viscous elastics. When the weight ratio of S_8 to TADA is 1 to 2, 1 to 3, and 1 to 5, the resulting copolymers are red translucent viscous elastics.

Preparation of poly(S_8/TAH): The polymerization was carried out by following the general method mentioned above with various weight ratios of TAH. The weight ratio of S_8 to TAH is 3 to 1, 2 to 1, 1 to 1, 1 to 2, and 1 to 3, respectively. When the weight ratio of S_8 to TAH is 3 to 1 and 2 to 1, partial S_8 precipitated after cooling down to room temperature resulting in heterogeneous copolymers. When the weight ratio of S_8 to TAH is 1 to 2 and 1 to 3, the resulting copolymers are red translucent copolymers.

Preparation of poly(S_8/TA): The polymerization was carried out by following the general method mentioned above with various weight ratios of TA. The weight ratio of S_8 to TA is 3 to 1, 2 to 1, 1 to 1, 1 to 2, and 1 to 3, respectively. After cooling down to room temperature, all products are yellow and waxy solid.

Preparation of poly(S_8/TAMe): The polymerization was carried out by following the general method mentioned above with various weight ratios of TAMe. The weight ratio of S_8 to TAMe is 3 to 1, 2 to 1, 1 to 1, 1 to 2, and 1 to 3, respectively. After cooling down to room temperature, poly(S_8/TAMe=3/1) is a yellow solid while the other products are a mixture of yellow solid and oil.

Preparation of poly(S_8/DIB): The preparation procedure for poly(S_8/DIB) under thermal condition is given below.[3]

Preparation of poly(S_8/DIB) at 120 °C: 500 g (1.95 mmol) of S_8 monomer was added into a Teflon vial, which was then heated by a metal heating block at a constant temperature of 120 °C. Yellow viscous liquid S_8 was obtained under magnetic stirring. A given amount of DIB was added into the molten S_8 liquid, the weight ratio of S_8 to DIB is 1 to 1 or 7 to 3, respectively. The reaction mixture was stirred at 120 °C for 2 hours to obtain homogeneity, and then cooled to room temperature to form a yellow solid.

Preparation of poly(S_8/DIB) at 160 °C: 500 g (1.95 mmol) of S_8 monomer was added into a Teflon vial, which was then heated by a metal heating block at a constant temperature of 120 °C. Yellow viscous liquid S_8 was obtained under magnetic stirring. A given amount of DIB was added into the molten S_8 liquid, the weight ratio of S_8 to DIB is 1 to 1 or 7 to 3, respectively. The reaction mixture was reacted at 160 °C for 12 hours. Then the product was cured in a vacuum oven at 140 °C for 12 h, yielding crimson solid.

3. Supplementary Figures

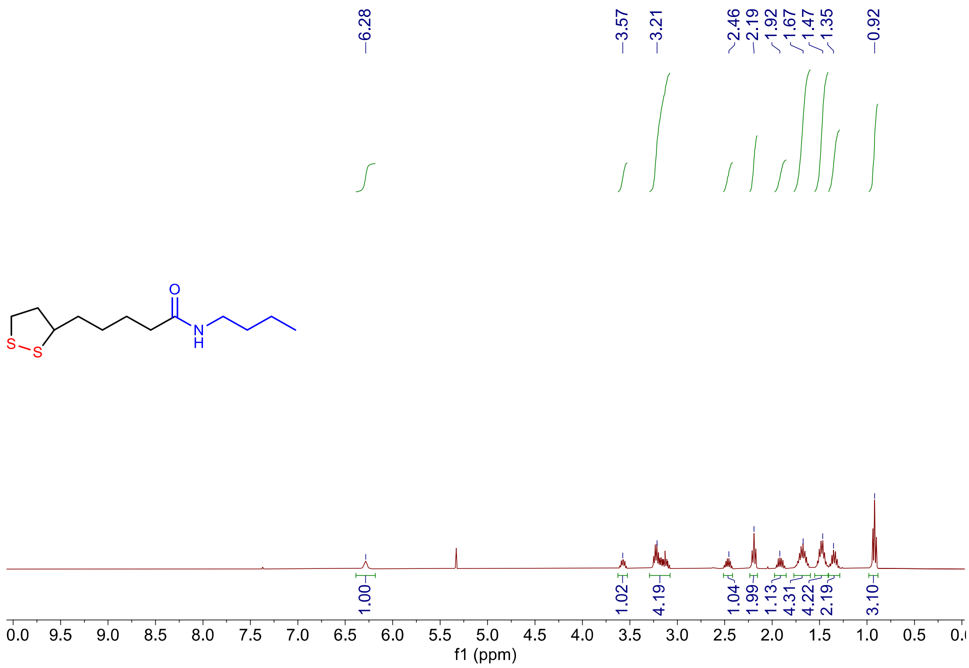

Supplementary Fig. 1 | ^{1}H NMR spectrum of compound TABA. ^{1}H NMR spectrum of TABA (CD_2Cl_2-d_2, 400 MHz, 298 K). The asterisked signals at δ 5.32 (^{1}H) are due to partially non-deuterated residues of CD_2Cl_2-d_2.

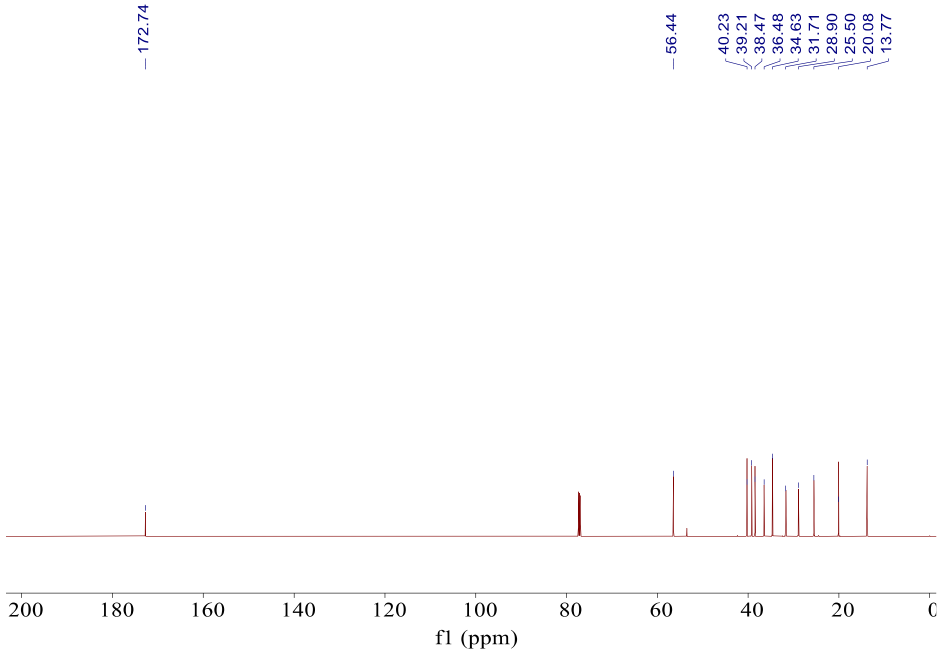

Supplementary Fig. 2 | ^{13}C NMR spectrum of compound TABA. ^{13}C NMR spectrum of compound TABA ($CDCl_3$-d_1, 151 MHz, 298 K).

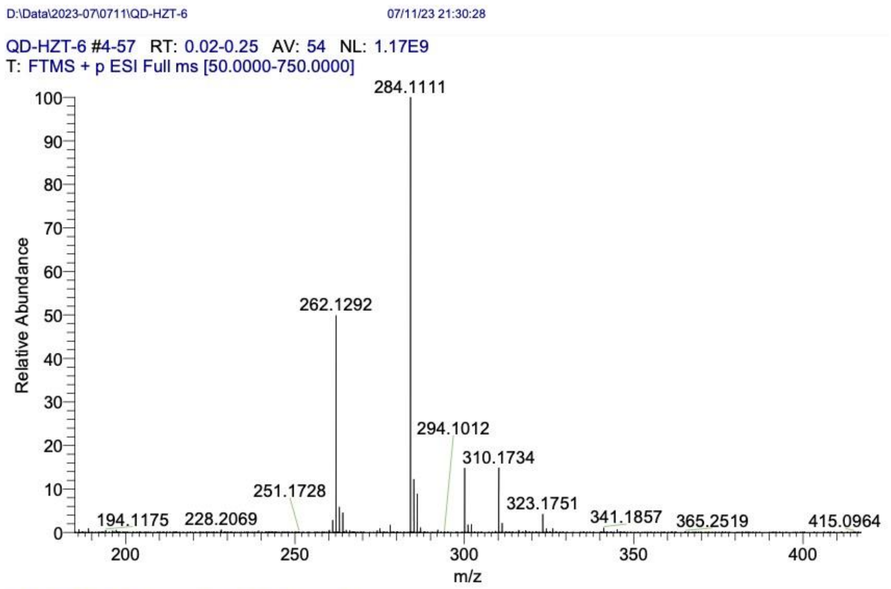

QD-HZT-6#26-58 RT: 0.11-0.25 AV: 33
T: FTMS + p ESI Full ms [50.0000-750.0000]
m/z= 260.38-286.33

m/z	Intensity	Relative	Theo. Mass	Delta (ppm)	Composition
262.1292	514739744.0	35.14	262.1294	-0.20	$C_{12} H_{24} O N S_2$
284.1111	1464970752.0	100.00	284.1113	-0.22	$C_{12} H_{23} O N Na S_2$

Supplementary Fig. 3 | High resolution mass spectrum of TABA. ESI-mass spectrum of compound TABA, $[M + H]^+$ calcd for $C_{12}H_{23}NOS_2$ + H: 262.1294, found 262.1292.

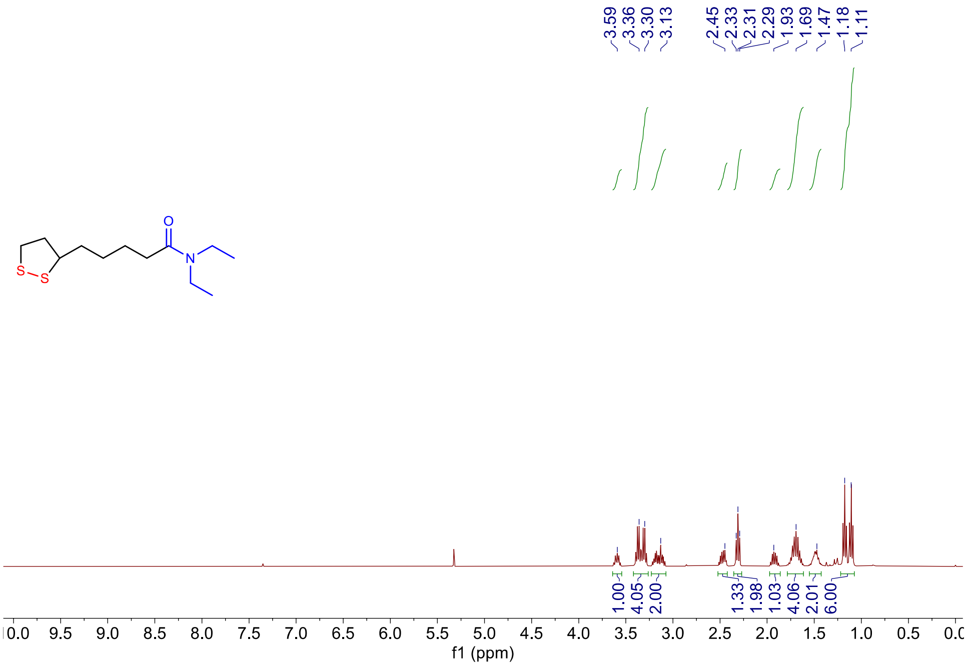

Supplementary Fig. 4 | ^{1}H NMR spectrum of compound TADA. ^{1}H NMR spectrum of TADA (CD_2Cl_2-d_2, 400 MHz, 298 K). The asterisked signals at δ 5.32 (^{1}H) are due to partially non-deuterated residues of CD_2Cl_2-d_2.

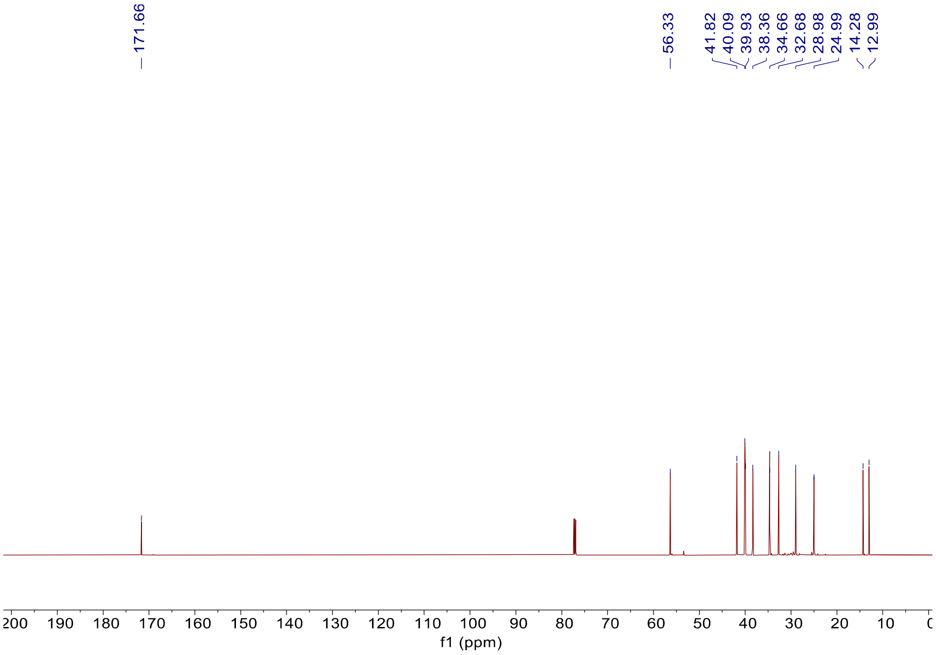

Supplementary Fig. 5 | ^{13}C NMR spectrum of compound TADA. ^{13}C NMR spectrum of compound TADA ($CDCl_3$-d_1, 151 MHz, 298 K).

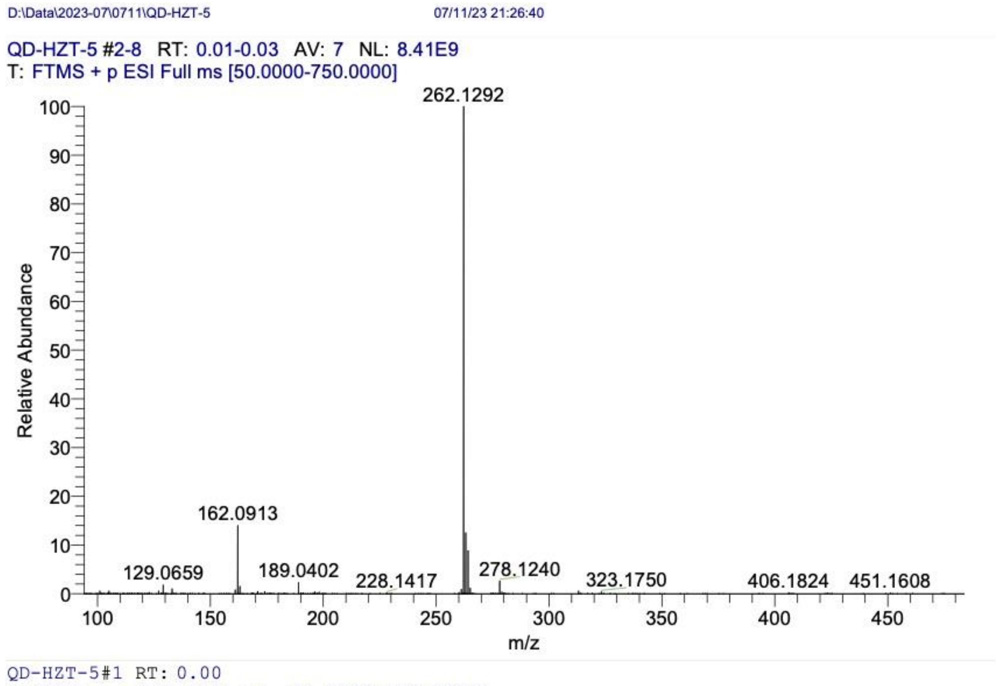

QD-HZT-5#1 RT: 0.00
T: FTMS + p ESI Full ms [50.0000-750.0000]

m/z	Intensity	Relative	Theo. Mass	Delta (ppm)	Composition
262.1292	9180653568.0	100.00	262.1294	-0.20	$C_{12}H_{24}ONS_2$

Supplementary Fig. 6 | High resolution mass spectrum of TADA. ESI-mass spectrum of compound TADA, $[M + H]^+$ calcd for $C_{12}H_{23}NOS_2$ + H: 262.1294, found 262.1292.

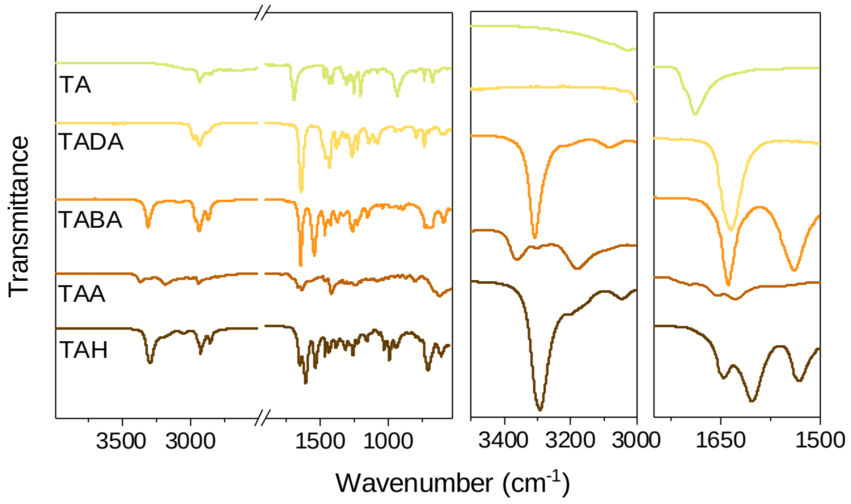

Supplementary Fig. 7 | ATR analysis of monomers. ATR data of TAA, TABA, TADA, TAH, TA monomer.

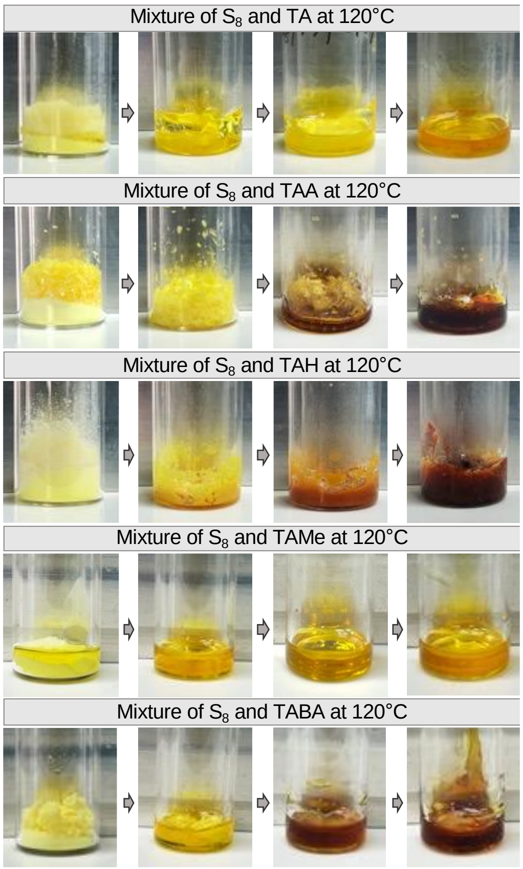

Supplementary Fig. 8 | Copolymerization procedure of S_8 and TA-based derivatives at 120°C. The weight ratio of S_8 to monomer is 1 to 1.

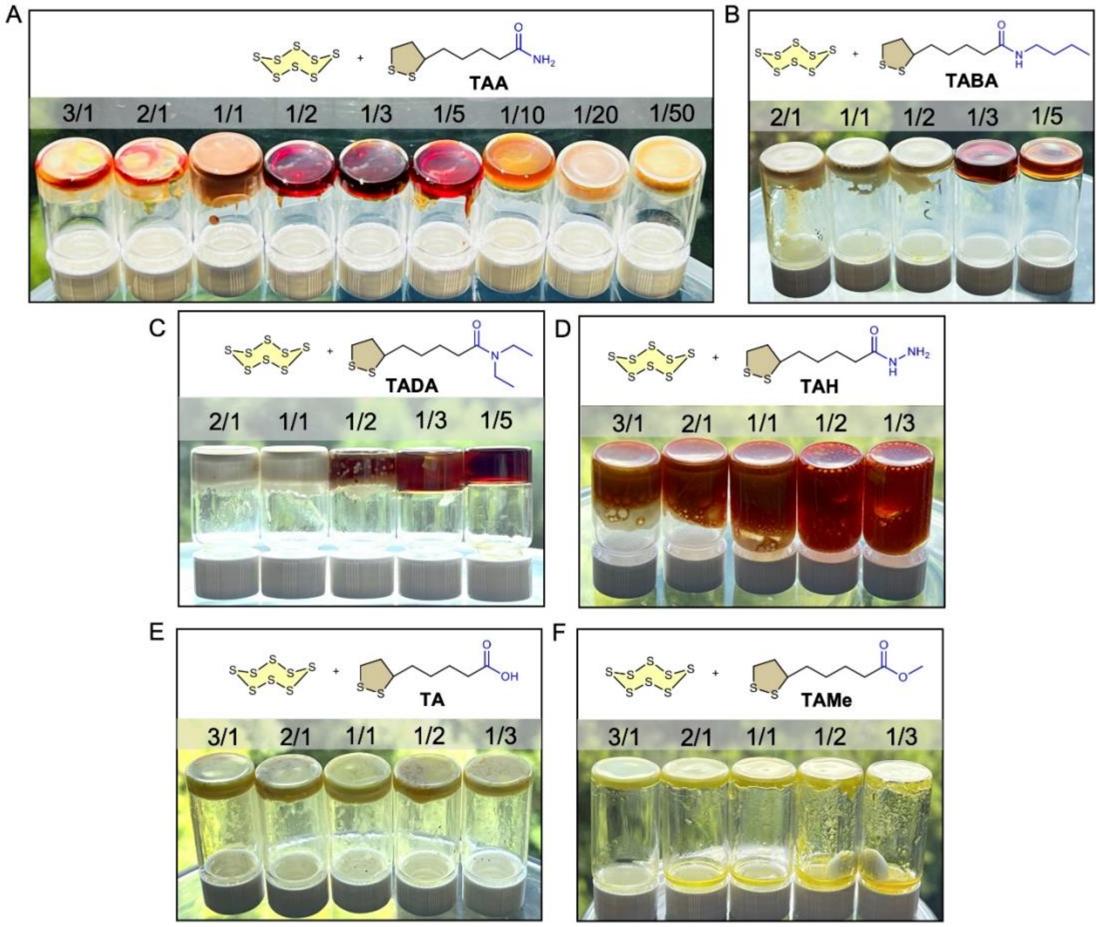

Supplementary Fig. 9 | Photographs of S_8 copolymers. (A) S_8 copolymers with various weight ratio of TAA monomer; (B) S_8 copolymers with various weight ratio of TABA monomer; (C) S_8 copolymers with various weight ratio of TADA monomer; (D) S_8 copolymers with various weight ratio of TAH monomer; (E) S_8 copolymers with various weight ratio of TA monomer; (F) S_8 copolymers with various weight ratio of TAMe monomer.

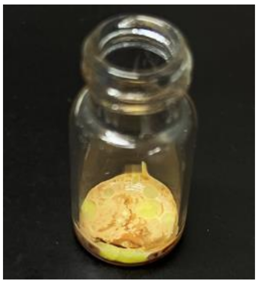

Supplementary Fig. 10 | Photograph of poly(S_8/TAA=3/1). Excess S_8 precipitate from the copolymer network.

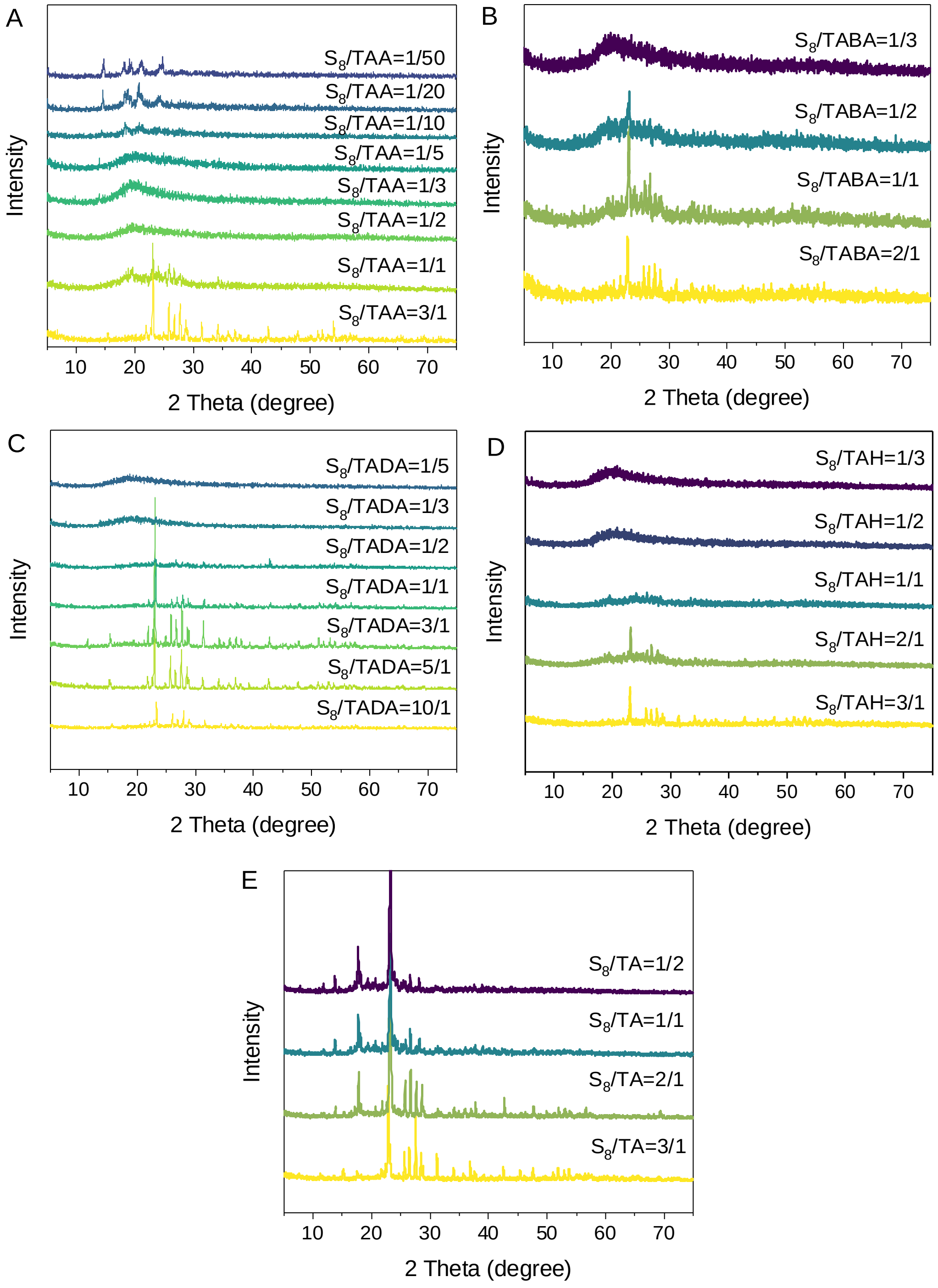

Supplementary Fig. 11 | XRD analysis. XRD patterns of (A) S_8 copolymers with various weight ratios of TAA monomer; (B) S_8 copolymers with various weight ratios of TABA monomer; (C) S_8 copolymers with various weight ratios of TADA monomer; (D) S_8 copolymers with various weight ratio of TAH monomer; (E) S_8 copolymers with various weight ratio of TA monomer.

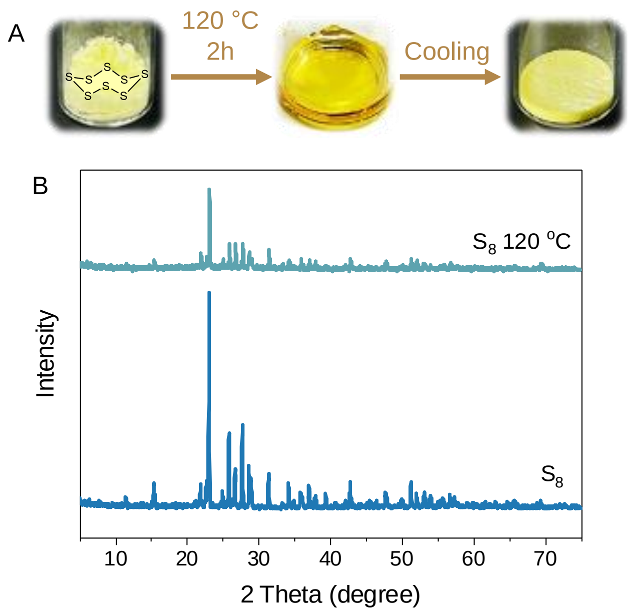

Supplementary Fig. 12 | (A) Polymerization process of S_8 at 120 °C; (B) XRD patterns of S_8 and its product after heating.

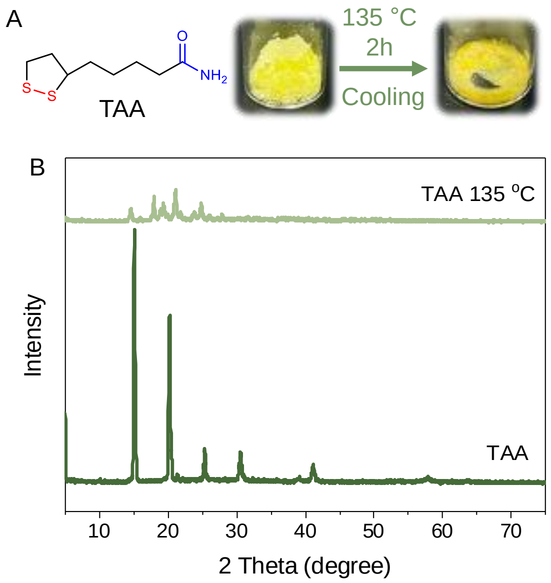

Supplementary Fig. 13 | (A) Polymerization process of TAA at 135 °C; (B) XRD patterns of TAA and its product after heating.

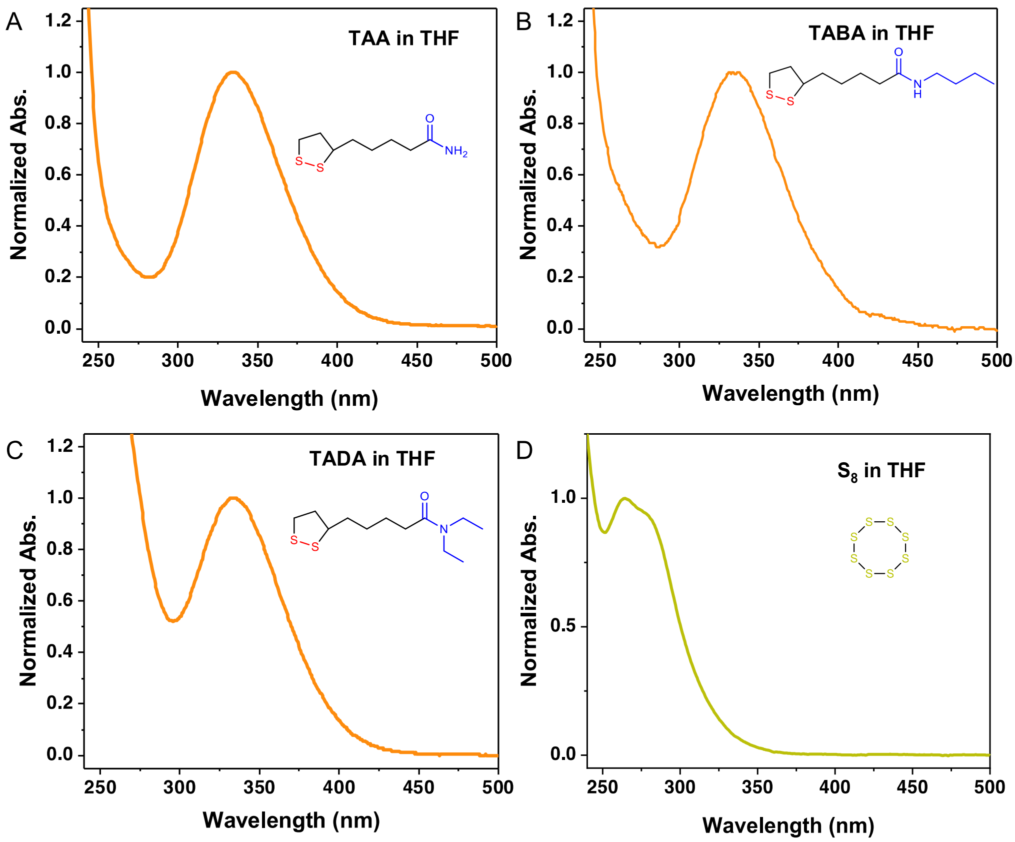

Supplementary Fig. 14 | UV-vis spectra analysis. The UV-vis spectra of the (A) TAA, (B) TABA, (C) TADA, and (D) S_8 monomers were measured in tetrahydrofuran (THF) solvent at room temperature.

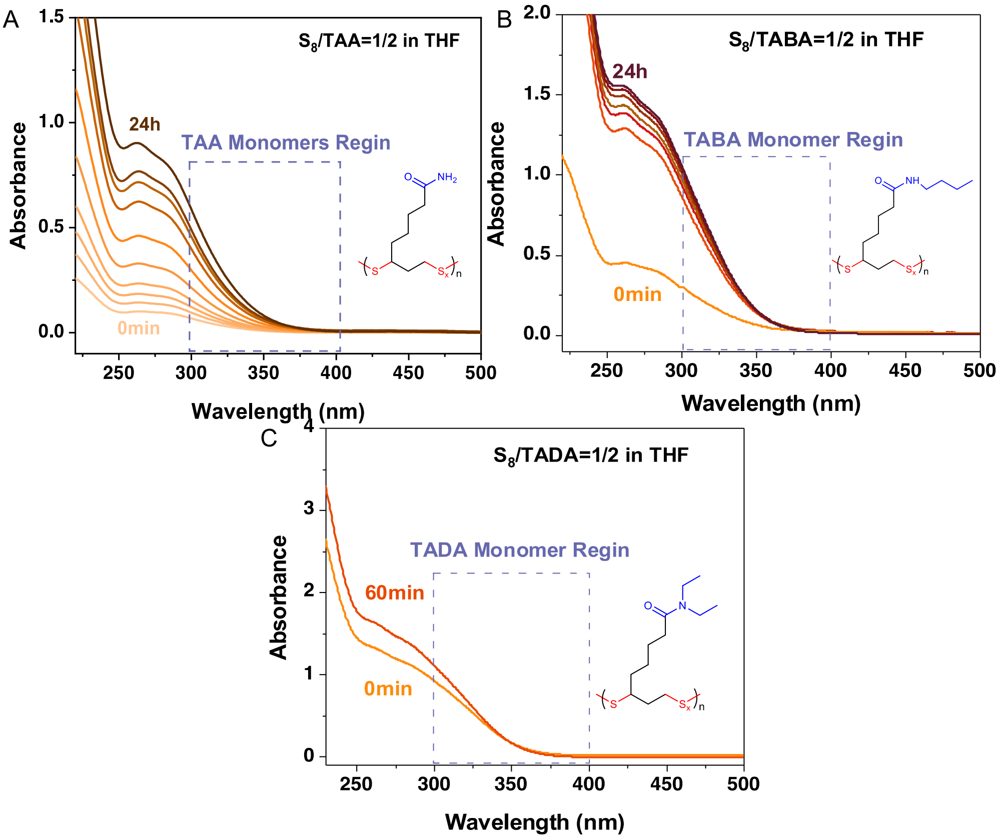

Supplementary Fig. 15 | UV-vis spectra analysis. The UV-vis spectra of the (A) poly(S_8/TAA=1/2), (B) poly(S_8/TABA=1/2), and (C) poly(S_8/TADA=1/2) were measured in tetrahydrofuran (THF) solvent at room temperature.

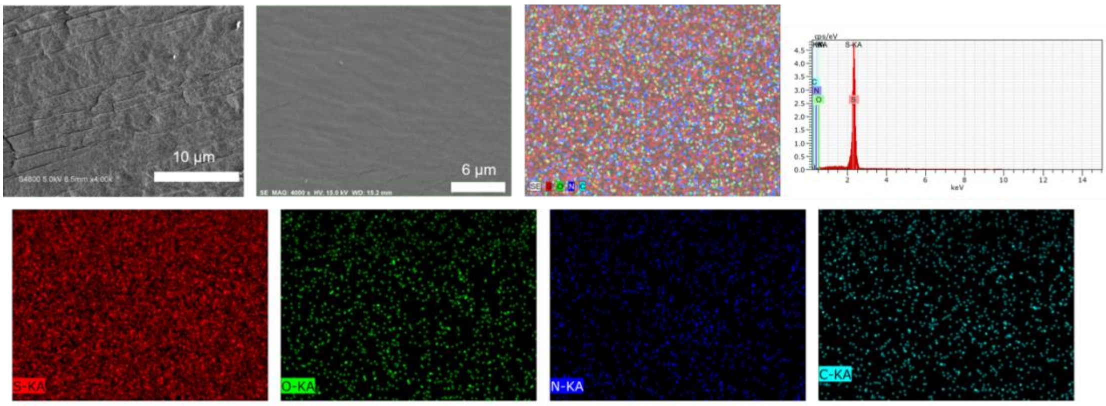

Supplementary Fig. 16 | SEM analysis. The morphology and element distribution of poly(S_8/TAA=1/2).

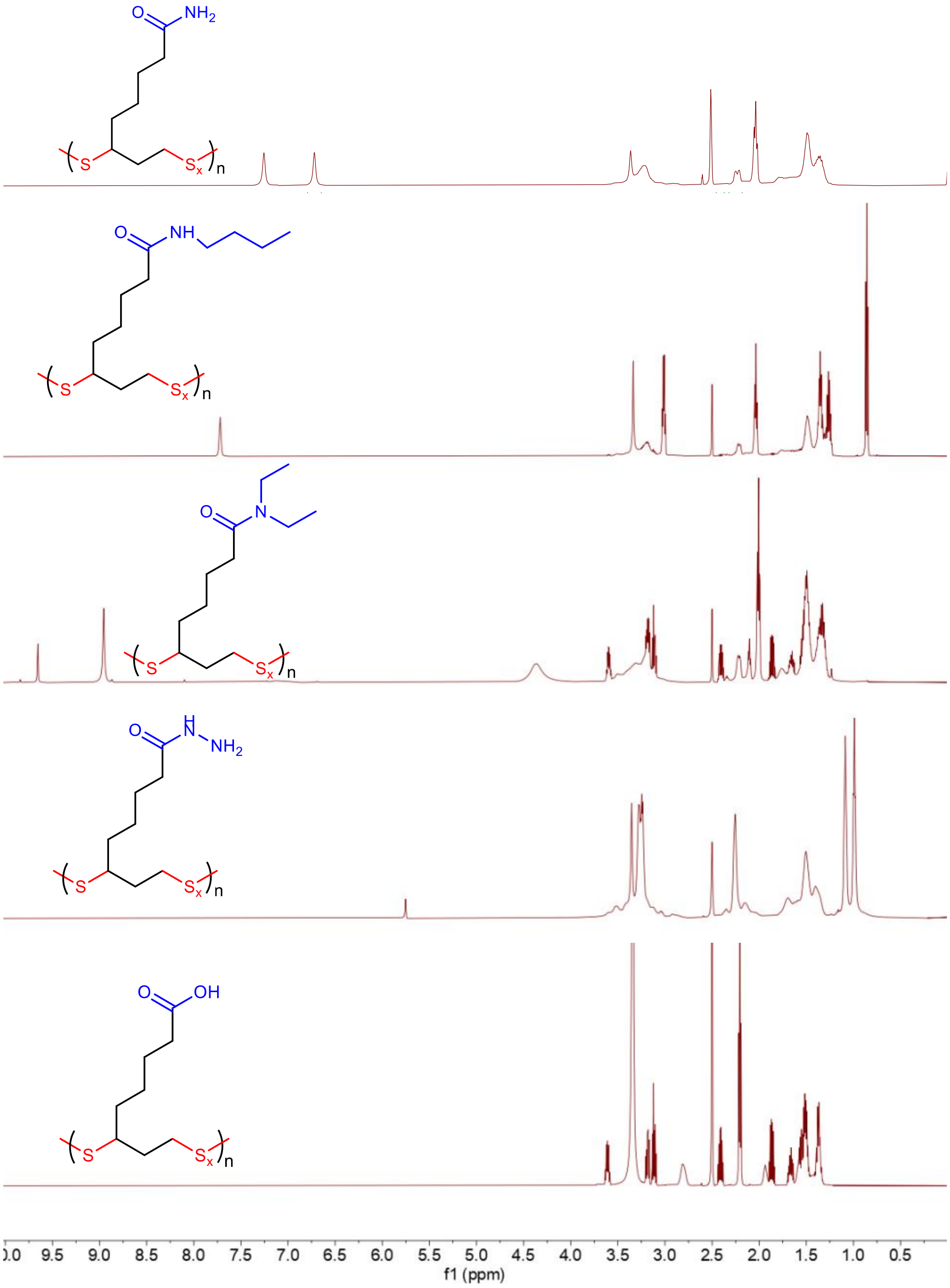

Supplementary Fig. 17 | ^{1}H NMR spectrum of copolymers. ^{1}H NMR spectrum of copolymers with various disulfide monomers, the weight ratio of S_8 to monomer is 1 to 2 (DMSO-d_6, 400 MHz, 298 K).

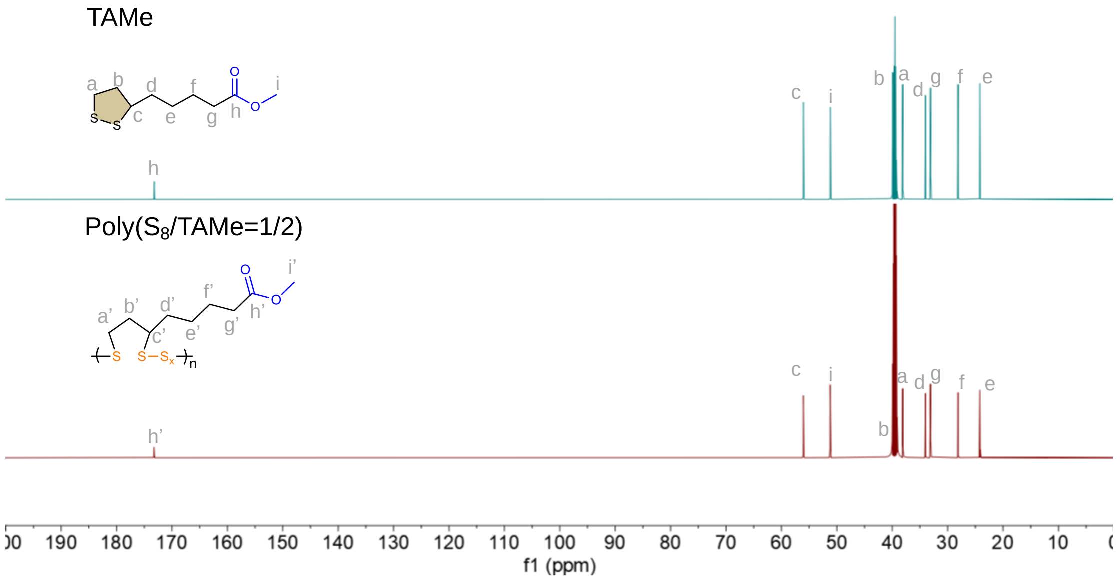

Supplementary Fig. 18 | ^{13}C NMR spectrum of compounds. ^{13}C NMR spectrum of compound TAMe and poly(S_8/TAMe) (DMSO-d_6, 151 MHz, 298 K).

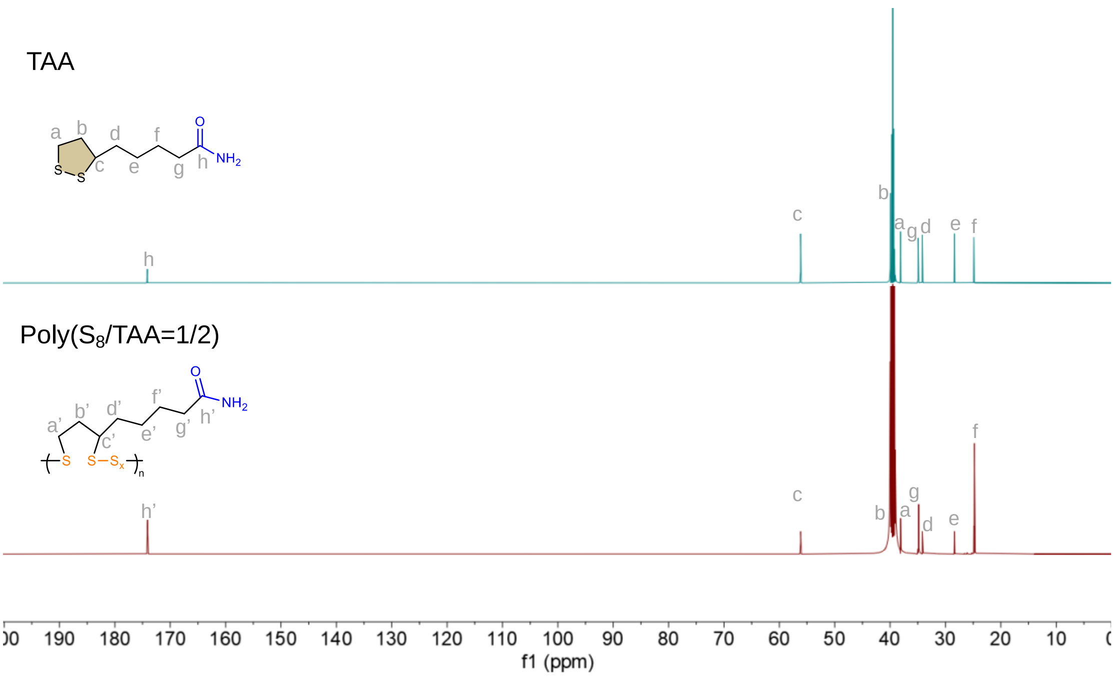

Supplementary Fig. 19 | ^{13}C NMR spectrum of compounds. ^{13}C NMR spectrum of compound TAA and poly(S_8/TAA) (DMSO-d_6, 151 MHz, 298 K).

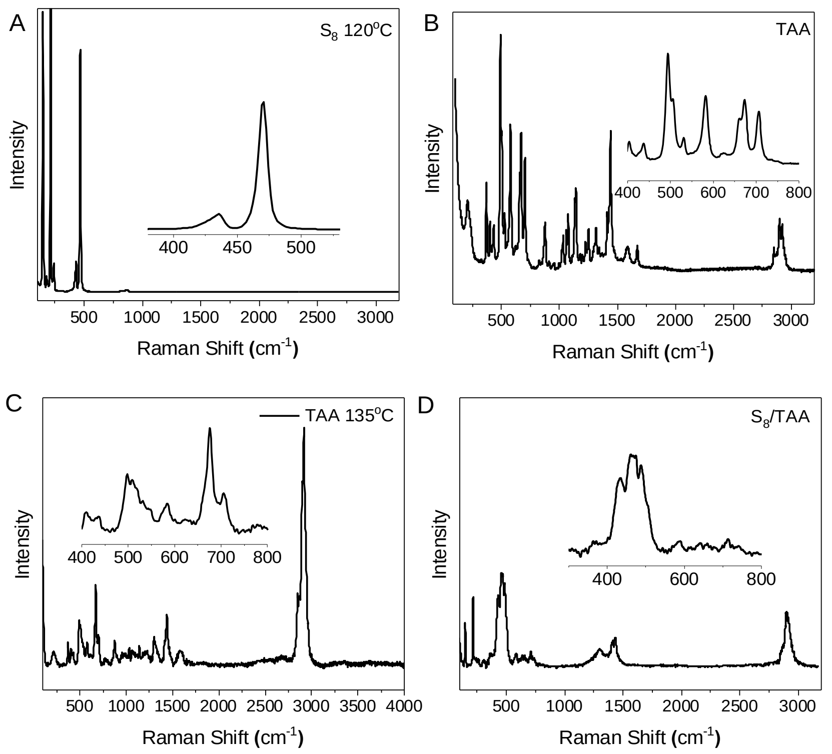

Supplementary Fig. 20 | Raman analysis. Raman spectra of (A) element sulfur after heating; (B) TAA monomer and its product after heating (C); (D) poly(S_8/TAA=1/3) copolymer.

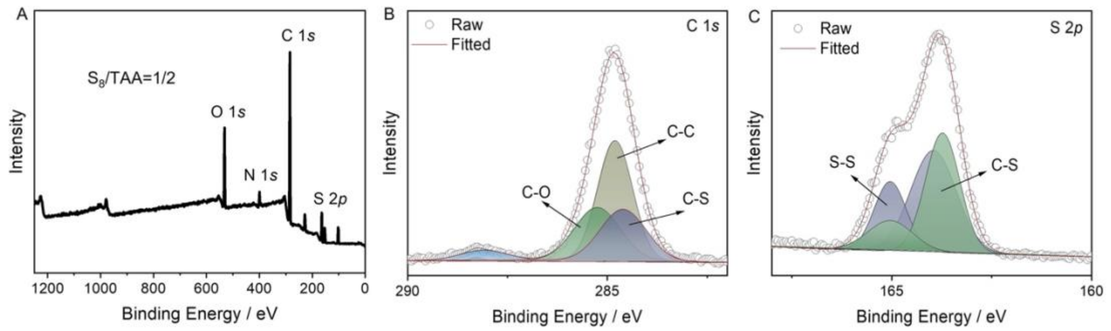

Supplementary Fig. 21 | XPS analysis. (A) XPS survey scan, (B) C 1*s* XPS spectrum, and (C) S 2*p* XPS spectrum of the product of poly(S_8/TAA=1/2).

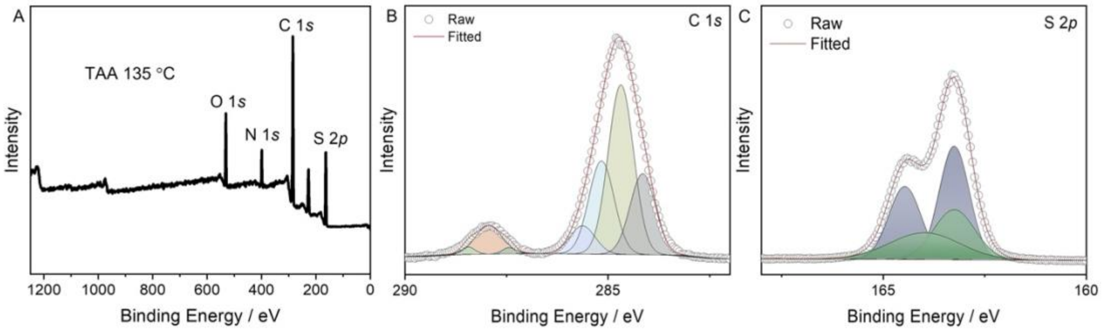

Supplementary Fig. 22 | XPS analysis. (A) XPS survey scan, (B) C 1*s* XPS spectrum, and (C) S 2*p* XPS spectrum of the product of TAA after heating.

A

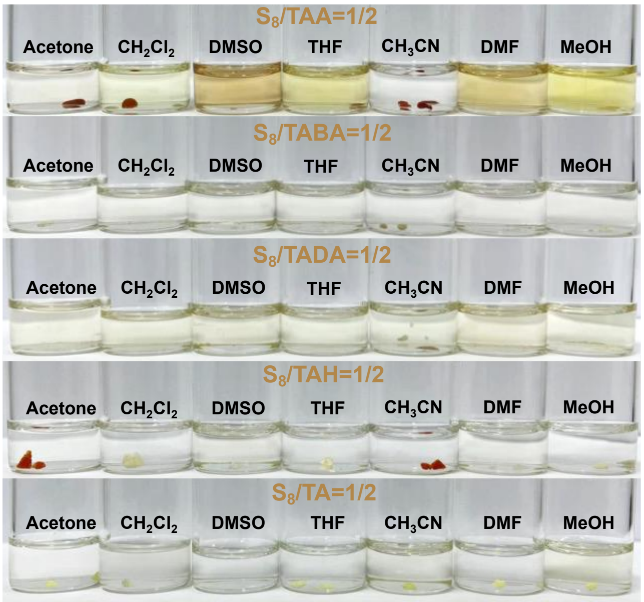

B

	S_8/TAA	S_8/TABA	S_8/TADA	S_8/TAH	S_8/TA
Acetone	X	X	X	X	X
CH_2Cl_2	X	√	√	X	X
DMSO	√	√	√	√	√
THF	△	△	△	X	△
CH_3CN	X	X	X	X	X
DMF	√	√	√	√	X
MeOH	△	△	△	△	△

Supplementary Fig. 23 | Solubility tests of different copolymer samples. Solubility test of the resulting copolymers. (A) Photographs of the polymer samples soaked in different solvents for 24 h; (B) Tables of the relationships between solvent solubility and copolymers. ("√" means totally soluble; "Δ" means swelling; "×" means insoluble). CH_2Cl_2: dichloromethane, DMSO: dimethyl sulfoxide, THF: tetrahydrofuran, CH_3CN: acetonitrile, DMF: N,N-dimethylformamide, MeOH: methanol.

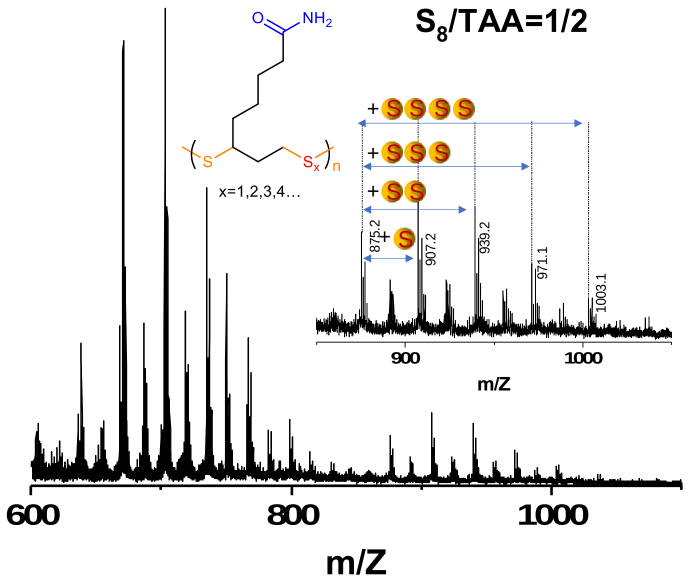

Supplementary Fig. 24 | MALDI-TOF-MS of poly(S_8/TAA=1/2) spectra. The polymer was dissolved in THF solvent.

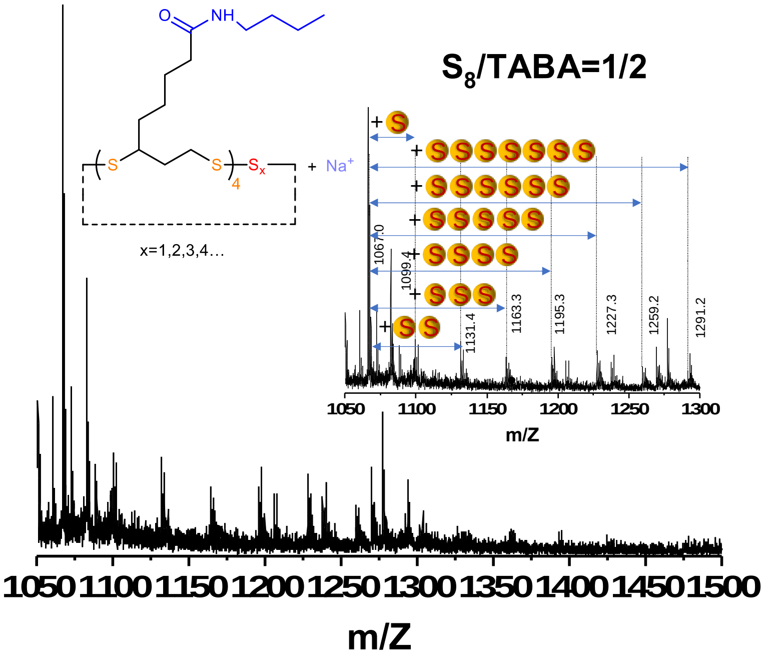

Supplementary Fig. 25 | MALDI-TOF-MS of poly(S_8/TABA=1/2) spectra. The polymer was dissolved in THF solvent.

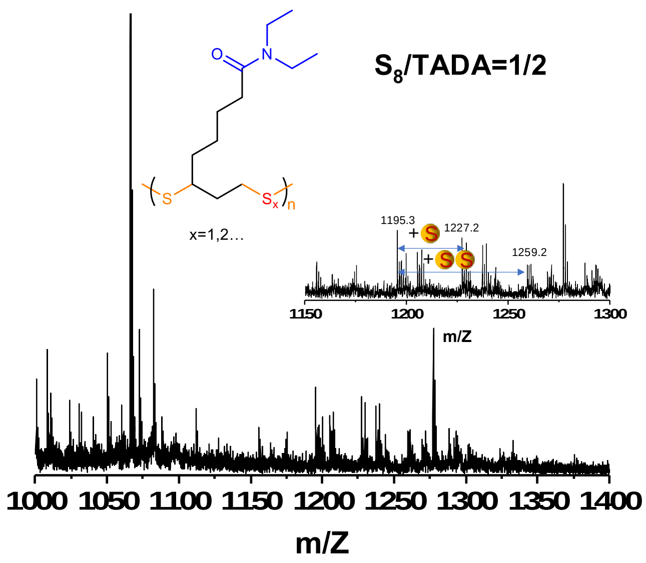

Supplementary Fig. 26 | MALDI-TOF-MS of poly(S_8/TADA=1/2) spectra. The polymer was dissolved in THF solvent.

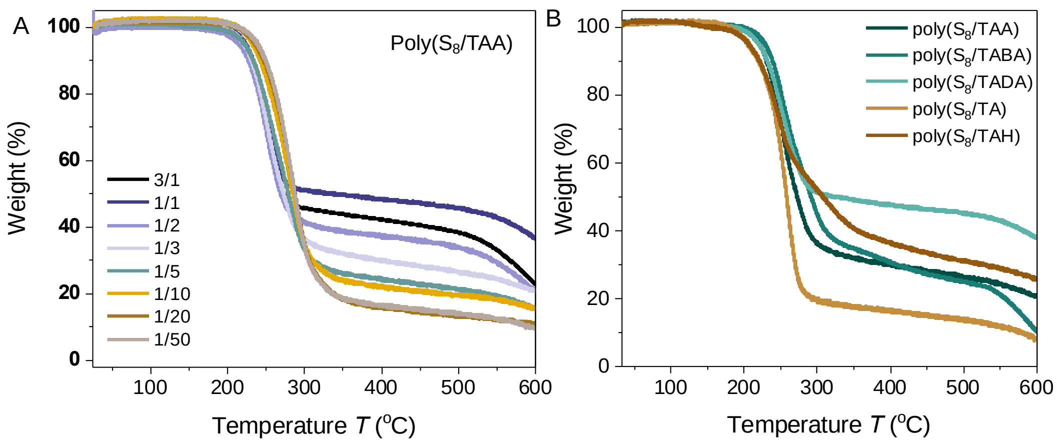

Supplementary Fig. 27 | TGA analysis. (A) poly(S_8/TAA) with various weight ratios of TAA. The first stage is due to the thermal decomposition of organic cyclic disulfides. The observation of the second stage is attributed to the inorganic sulfur in the product. (B) Sulfur-rich copolymers with various monomers. TGA data for polymers (room temperature to 600 °C in N_2) shows good thermostability with similar decomposition temperatures over 200 °C.

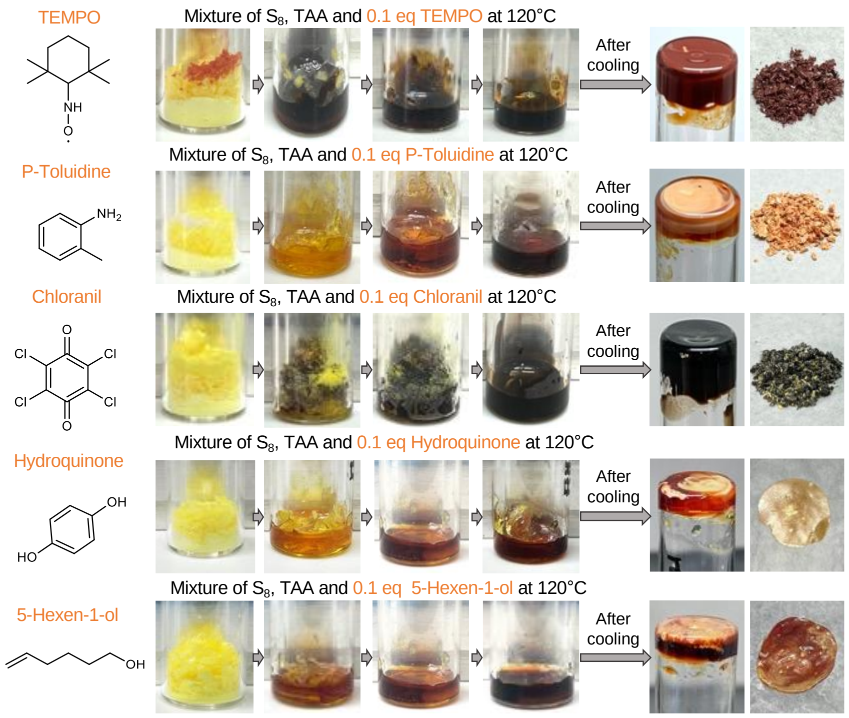

Supplementary Fig. 28 | Additional experiments to test the effect of radical quenchers/scavengers in the copolymerization system.

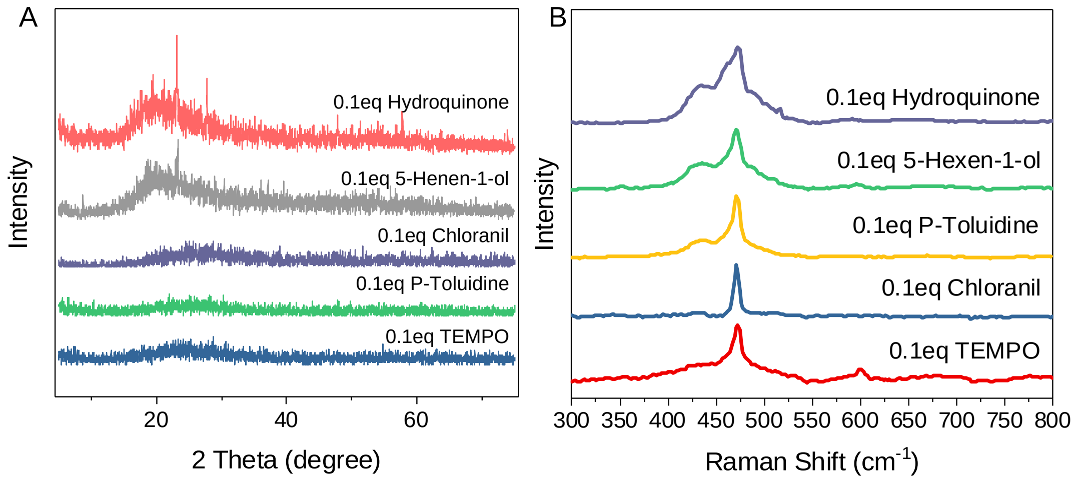

Supplementary Fig. 29 | XRD and Raman analysis. (A) XRD patterns and (B) Raman spectra of poly(S_8/TAA=1/1) with 0.1 eq various radical trapping agents, polymerization inhibitors, or sulfur radical quencher.

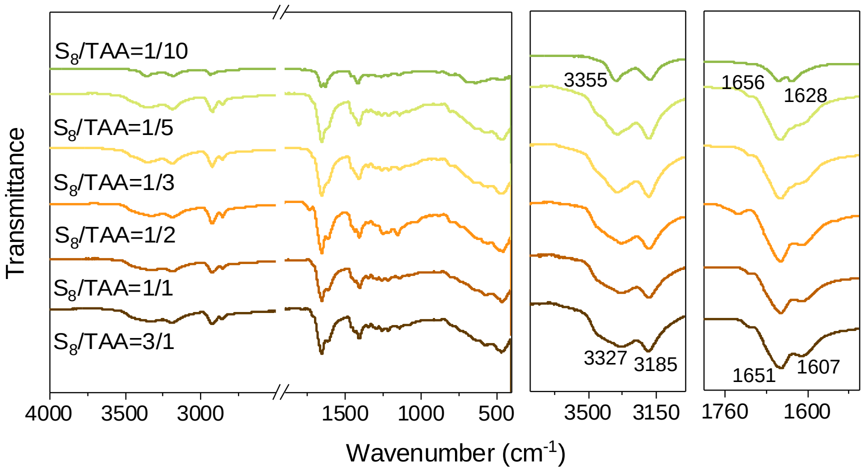

Supplementary Fig. 30 | ATR analysis. ATR data of poly(S_8/TAA) with various amounts of TAA monomer, the weight ratio of S_8 to TAA ranging from 1 to 10 to 3 to 1.

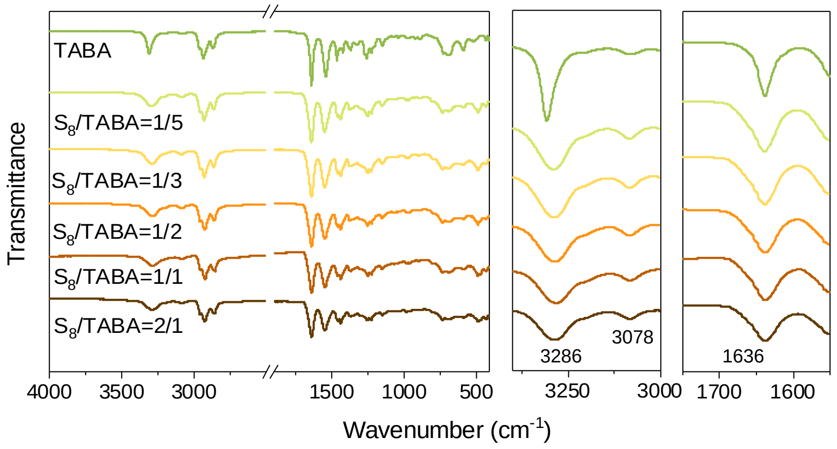

Supplementary Fig. 31 | ATR analysis. ATR data of poly(S_8/TABA) with various amounts of TABA monomer, the weight ratio of S_8 to TABA ranging from 1 to 5 to 2 to 1.

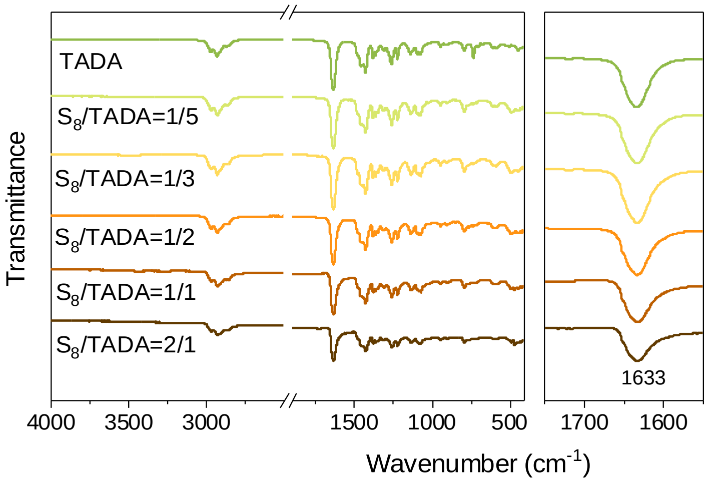

Supplementary Fig. 32 | ATR analysis. ATR data of poly(S_8/TADA) with various amounts of TADA monomer, the weight ratio of S_8 to TADA ranging from 1 to 5 to 2 to 1.

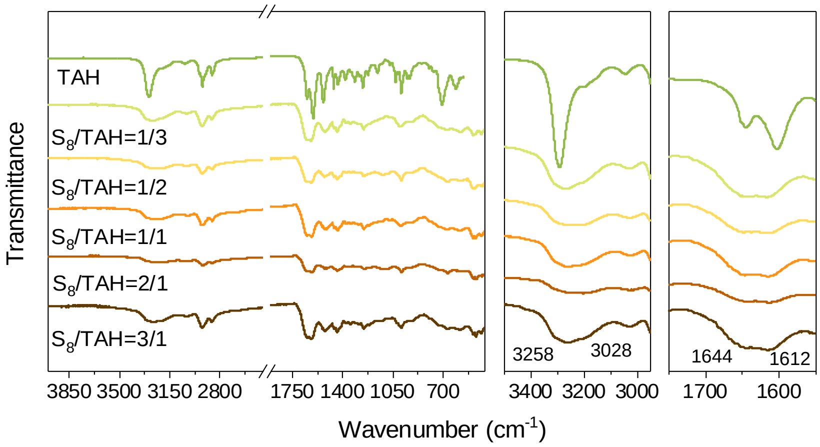

Supplementary Fig. 33 | ATR analysis. ATR data of poly(S_8/TAH) with various amounts of TAH monomer, the weight ratio of S_8 to TAH ranging from 1 to 3 to 3 to 1.

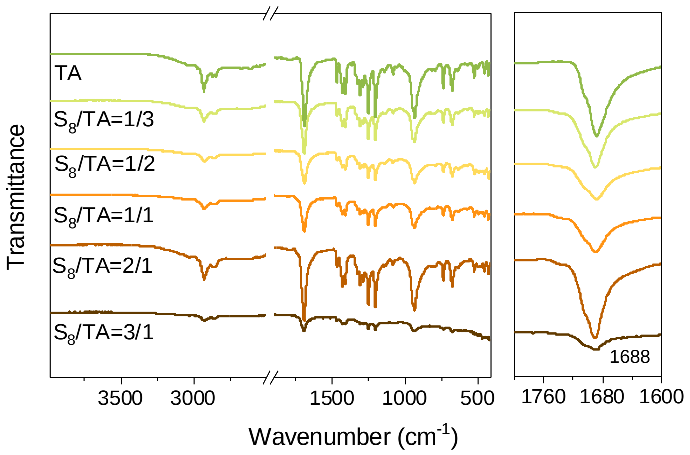

Supplementary Fig. 34 | ATR analysis. ATR data of poly(S_8/TA) with various amounts of TA monomer, the weight ratio of S_8 to TA ranging from 1 to 3 to 3 to 1.

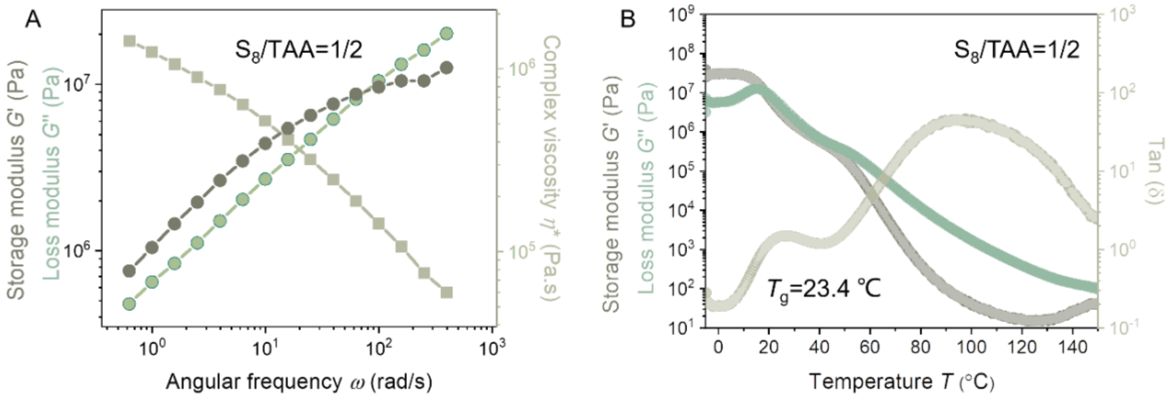

Supplementary Fig. 35 | Rheological curves of poly(S_8/TAA). (A) Frequency-dependence of storage moduli (*G*') and loss moduli (*G*'') measured at 25 °C; (B) Temperature-dependence of *G*' and *G*" at 1 Hz.

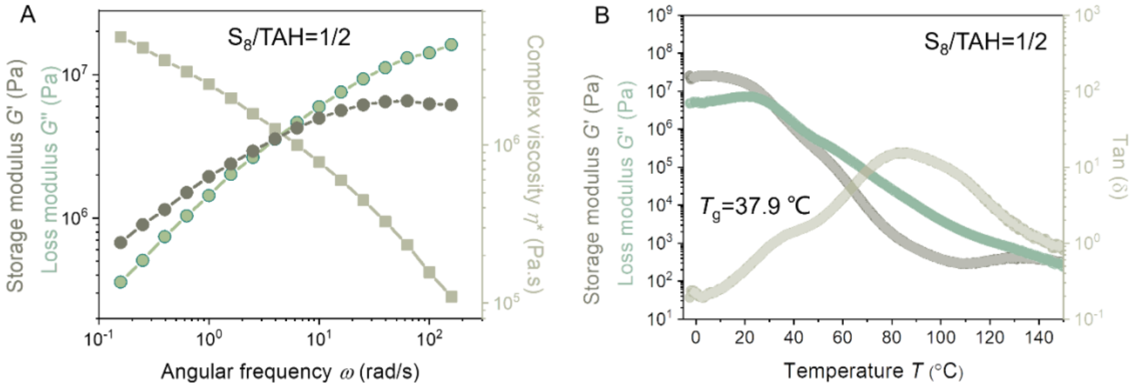

Supplementary Fig. 36 | Rheological curves of poly(S_8/TAH=1/2). (A) Frequency-dependence of storage moduli (*G*') and loss moduli (*G*'') measured at 25 °C; (B) Temperature-dependence of *G*' and *G*" at 1 Hz.

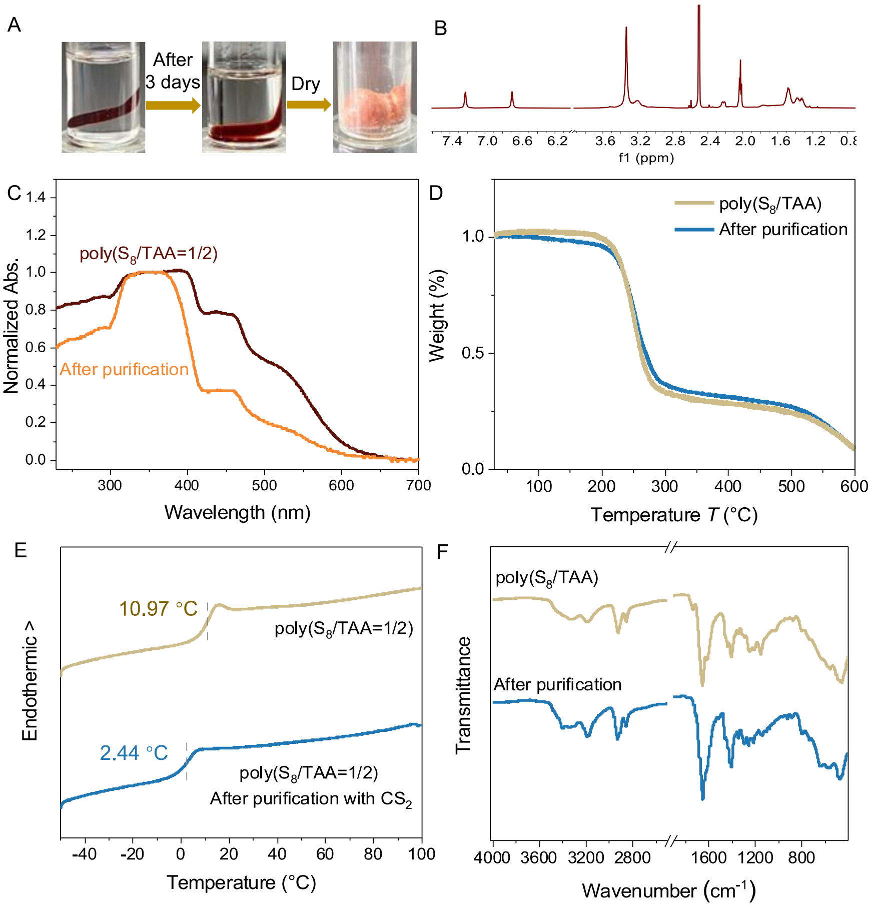

Supplementary Fig. 37 | Purification with CS_2. (A) The images of the purification process. (B) ^{1}H NMR spectrum, (C) Solid-state UV-Vis spectra, (D) TGA analysis, (E) DSC analysis, and (F) ATR analysis of the poly(S_8/TAA=1/2) after purification.

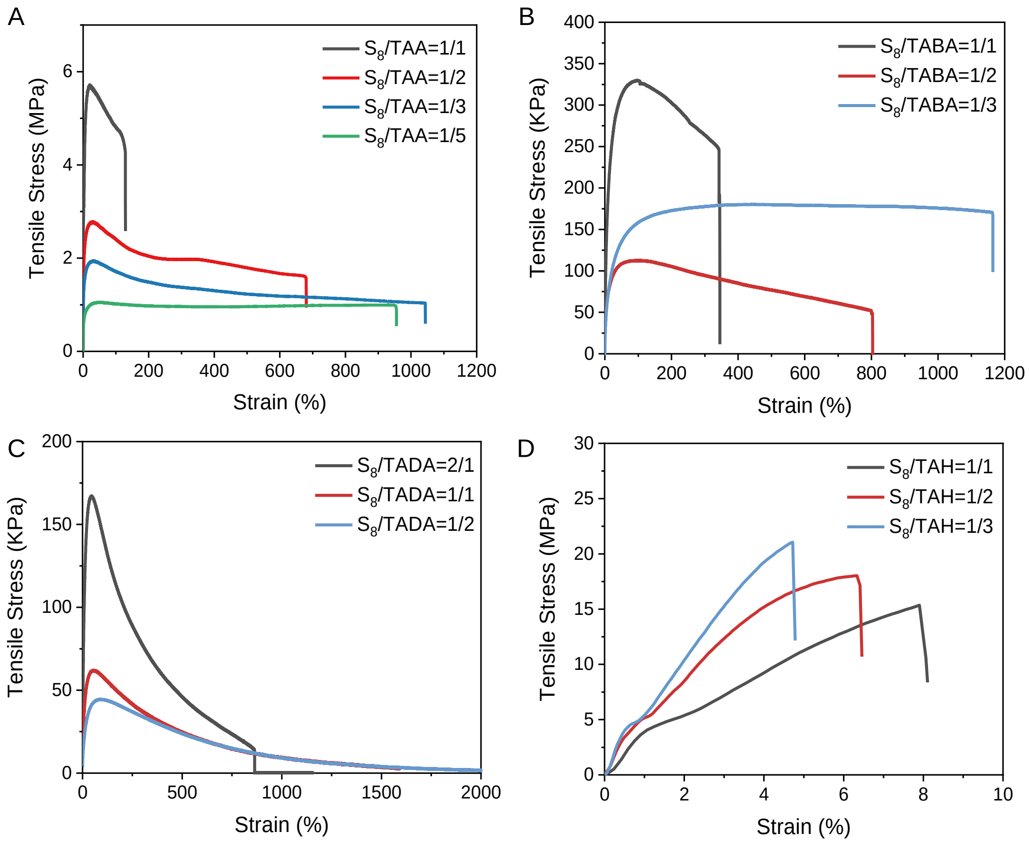

Supplementary Fig. 38 | Stress-strain curves of copolymers. (A-D) Stress-strain curves of the various copolymers. The measurement was carried out with a strain rate of 50 mm/min at room temperature.

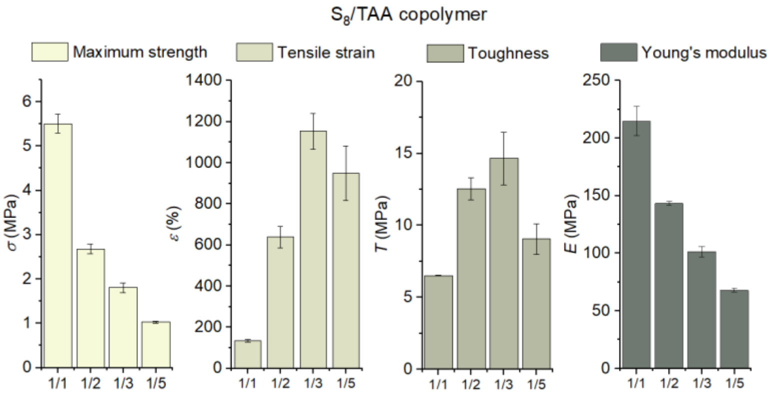

Supplementary Fig. 39 | Summary of the mechanical properties of poly(S_8/TAA). The weight ratio of S_8 to TAA is 1/1, 1 to 2, 1 to 3, and 1 to 5, respectively. Measurements were carried out in triplicate (n = 3), error bars show the standard deviation of the replicate measurements, the error bar centers are the means of the replicate measurements.

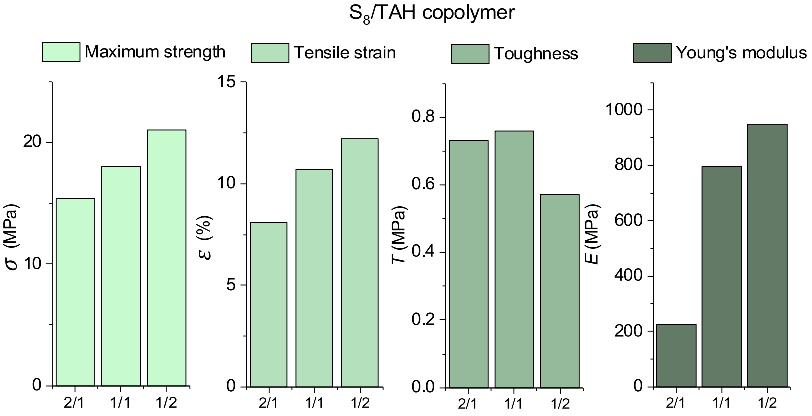

Supplementary Fig. 40 | Summary of the mechanical properties of poly(S_8/TAH). The weight ratio of S_8 to TAH is 2 to 1, 1 to 1, and 1 to 2, respectively.

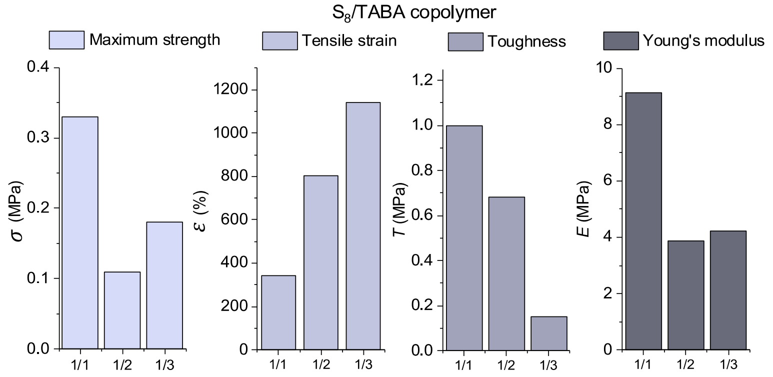

Supplementary Fig. 41 | Summary of the mechanical properties of poly(S_8/TABA). The weight ratio of S_8 to TABA is 1 to 1, 1 to 2, and 1 to 3, respectively.

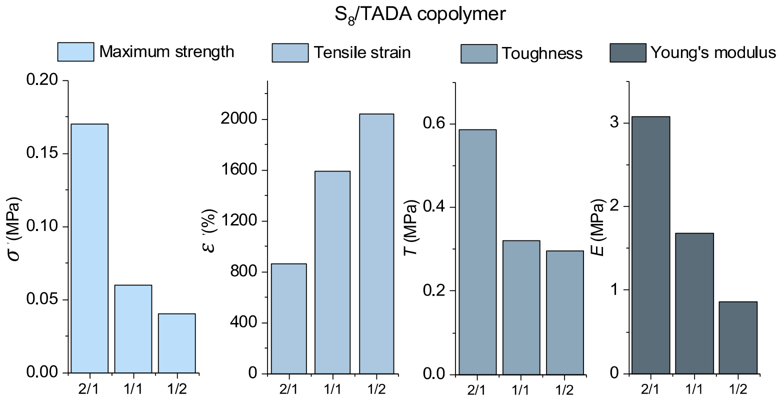

Supplementary Fig. 42 | Summary of the mechanical properties of poly(S_8/TADA). The weight ratio of S_8 to TADA is 2 to 1, 1 to 1, and 1 to 2, respectively.

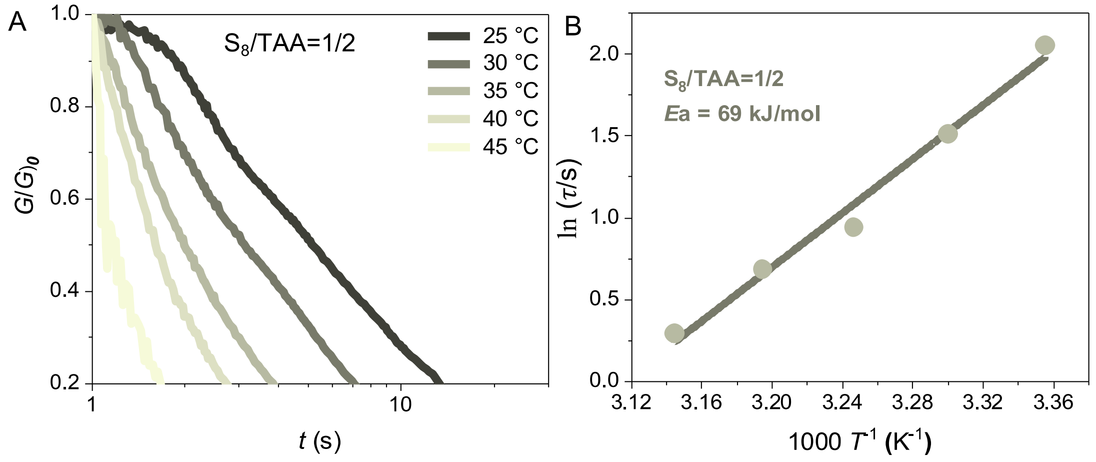

Supplementary Fig. 43 | Temperature-dependent stress-relaxation. (A) Normalized stress relaxation of poly(S_8/TAA=1/2) at a temperature range from 25 °C to 45 °C; (B) Plots for ln(τ) versus 1000/T of poly(S_8/TAA=1/2).

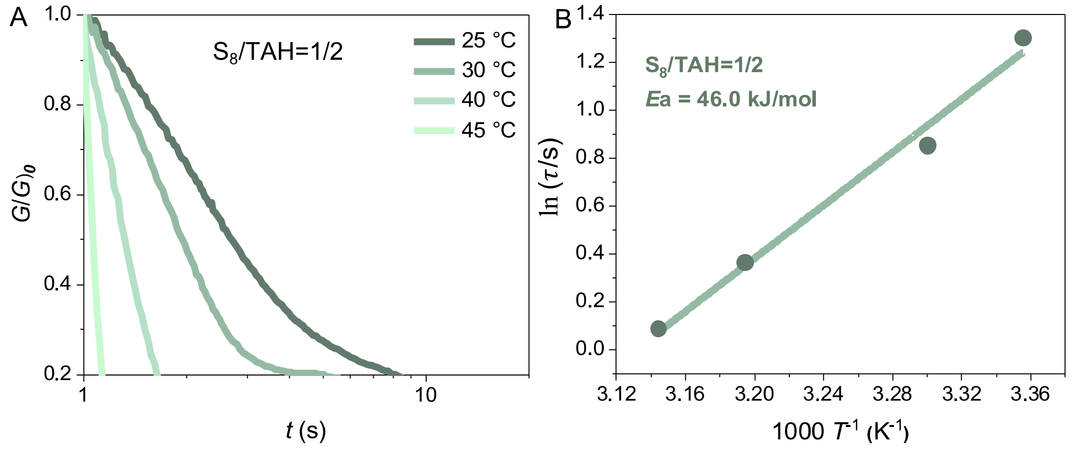

Supplementary Fig. 44 | Temperature-dependent stress-relaxation. (A) Normalized stress relaxation of poly(S_8/TAH=1/2) at a temperature range from 25 °C to 45 °C; (B) Plots for ln(τ) versus 1000/T of poly(S_8/TAH=1/2).

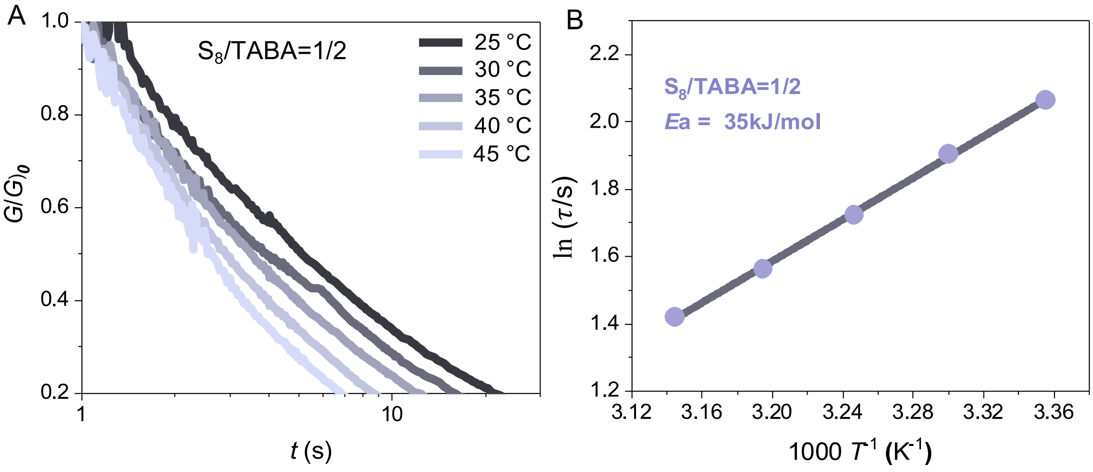

Supplementary Fig. 45 | Temperature-dependent stress-relaxation. (A) Normalized stress relaxation of poly(S_8/TABA=1/2) at a temperature range from 25 °C to 45 °C; (B) Plots for ln(τ) versus 1000/T of poly(S_8/TABA=1/2).

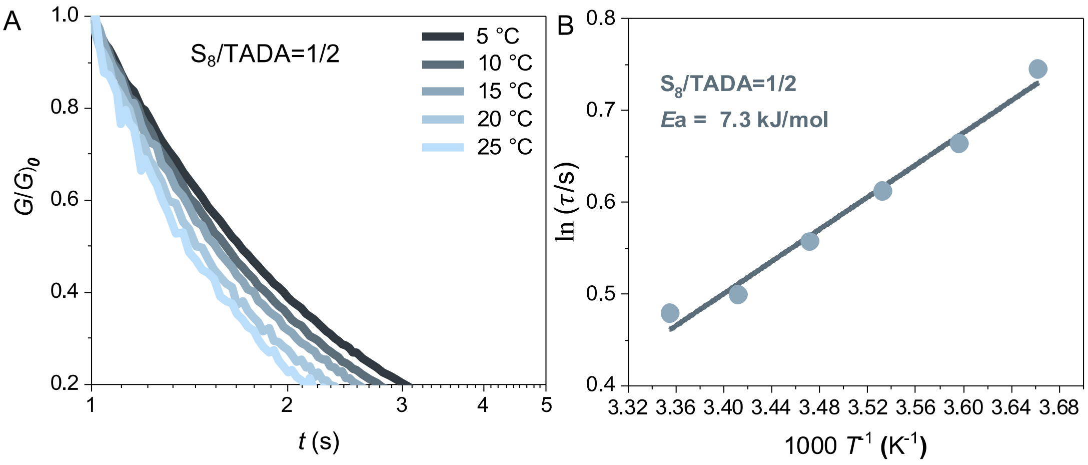

Supplementary Fig. 46 | Temperature-dependent stress-relaxation. (A) Normalized stress relaxation of poly(S_8/TADA=1/2) at a temperature range from 5 °C to 25 °C; (B) Plots for ln(τ) versus 1000/T of poly(S_8/TADA=1/2).

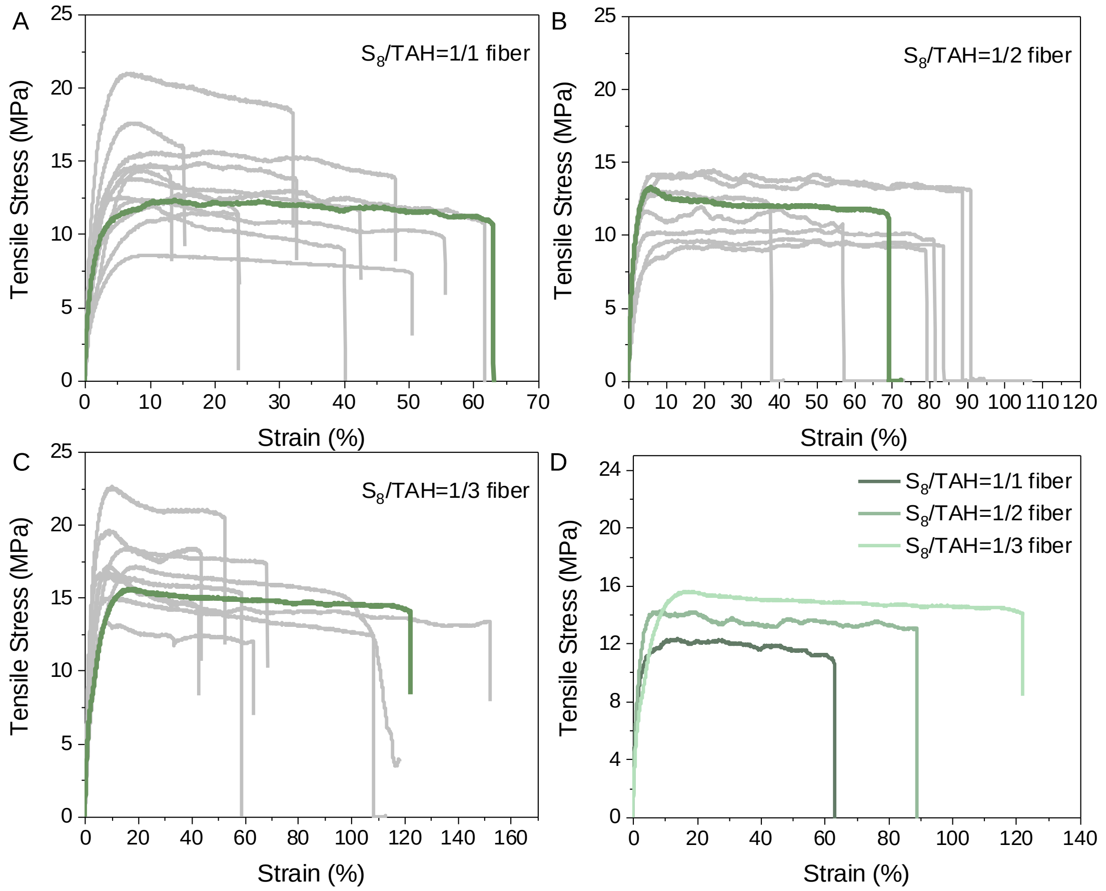

Supplementary Fig. 47 | Stress-strain curves of polymer filaments of poly(S_8/TAH). Stress-strain curves of (A) poly(S_8/TAH=1/1), (B) poly(S_8/TAH=1/2), (C) poly(S_8/TAH=1/3) filaments, and (D) their comparison. Strain rate is 10 mm/min.

Supplementary Table 1 | A summary of the mechanical properties of the poly(S_8/TAH) fiber. The weight ratio of S_8 to TAH is 1 to 1, 1 to 2, and 1 to 3, respectively.

S_8/TAH	Tensile strength (σ_{max}, Mpa)	Tensile strain (ε_b, %)	Young's modulus (E, MPa)	Toughness (U_T, MJ·m^{-3})
1/1	16.31 ± 3.04	44.29 ± 16.35	761.28 ± 72.03	6.56 ± 0.80
1/2	12.86 ± 2.33	78.39 ± 13.90	880.38 ± 87.82	9.36 ± 2.31
1/3	18.58 ± 2.69	82.32 ± 27.02	911.84 ± 56.75	16.55 ± 3.25

Supplementary Table 2 | Summary of recent research on sulfur-rich polymeric materials.

Year	Crosslinker	Tensile strength (Mpa)	Tensile strain (%)	Toughness (MJ/m^3)	Young's modulus(Mpa)	Application
2013[4]	DIB(10%-50%)	8.69	6.65	0.31	262; 190; 450; 330	Li–S battery cathodes
2015[5]	DIB(30%)	17.5	2.8	0.47	1390	Healable Optical Materials
2016[6]	TIB(30%-50%)	19.50; 9.43	1.63; 2.33	0.27; 0.17	1740; 1100	Infrared Optical Materials
	DIB(30%-50%)	2.33; 10.10	228.33; 1.27	3.61; 0.11	430; 1210	
2017[7]	MMA-POSS	22	3.7	N.A.	N.A.	Mid-Infrared materials
	DIB(20%)	0.23	72.23		14.74	
2017[8]	DVB(5%)+DIB(15%)	2.18	56.99	low toughness	76.84	Optical Lense
	DVB(15%)+Sty(5%)	2.52	44.06		42.37	
2017[9]	DA(10%-30%)	0.3; 0.5; 0.55	47.67;71.46; 53.62	N.A.	1.3; 2.1; 6.0	Thermal Insulators
	DCPD(25%)+Linseed Oil(25%)	4.5	1.25	N.A.	495	
2019[10]	DCPD(25%)+EDGMA(25%)	1.50	2.75	N.A.	120	Brittle Materials
	DCPD(25%)+Limonene(25%)	4.50	5.45	N.A.	115	
2019[11]	Alkenes+catalysts	N.A.	N.A.	N.A.	N.A.	Metal Uptake
2019[12]	TTS(30%-50%)	7.0; 6.8	0.9; 1.5	N.A.	N.A.	N.A.
2019[13]	Lignin(10%-20%)	2.0; 1.4; 1.55	0.04; 0.03; 0.08	N.A.	N.A.	Recyclable Materials
2019[14]	Allyl Glycldyl Ether(30%-50%)	13.55; 60.44	16.7; 3.0	N.A.	N.A.	Shape-memory Materials
2019[15]	Liquid Metal+DIB(10-30vol%)	0.59; 0.74; 0.85	8.28; 9.94; 9.11	N.A.	12.35; 15.25; 17.57	Conductive Materials
2020[16]	Squalene(40%)+β -Myrcene(10%-30%)	1.4; 0.4; 0.1	270; 330; 440	N.A.	9.50; 0.67; 0.13	Elastic IR-transmitting Materials
2020[17]	MDI+span80	20.17; 9.64; 1.88; 0.59	11.85; 16.08; 42.06; 51.2	N.A.	N.A.	Shape-memory Materials
2020[18]	Cellulose(10%-20%)	5.00; 4.05; 3.80	0.90; 0.76; 1.15	N.A.	N.A.	N.A.
2020[19]	Linoleic acid(1-10%)	1.2; 1.8; 1.5	3.0; 6.0; 8.0	N.A.	N.A.	N.A.
2020[20]	Aryl Halides(20%)	3	32	N.A.	N.A.	N.A.
	SPU+MDI+Phenyl isocyanate	8.35	155.00	9.84	1.16	
2021[21]	SPU+MDI+Phenyl isocyanate+DIB	24.91	348.00	73.4	2.83	High Perfomance Plastic
	SPU+MDI+Phenyl isocyanate+PDMS	13.54	578.00	58.1	0.54	
2022[22]	DIB(Thermal Sythesis)	0.26	349.08	N.A.	0.92	Li-S battery
	DIB(Mechanol Sythesis)	3.98	44.44	N.A.	11.37	
2022[23]	span80+BADGE(BA25-BA100)	0.48; 1.51;	215.1; 171.05	47.12; 202.84 (J/m^3)	0.24; 1.91	Stretching Sensors
		6.36; 13.01	167.3; 113.28	782.65; 1273.36 (J/m^3)	116.91; 470.65	
2023[24]	BDDA+DMA	3.80	29.00	N.A.	N.A.	Reprocessable copolymers

DIB: m-diisopryl benzene
TIB: 1,3,5-Triisopropenylbenzene
MMA-POSS: multifunctional methacrylated polyhedral oligomeric silsesquioxane
DVB: Sivinylbenzene
Sty: Styrene
DA: Diallyl disulfide
DCPD: Dicyclopentadiene
EDGMA: Ethylene glycol dimethylacrylate
TTS: Tretraallyltyrosine
Span80: Sorbitan oleate
MDI: 4,5'-diphenylmethane diisocyanate
SPU: Sulfur-based polyurethane
PDMS: Polydimethylsiloxane
DMA: N,N-dimethylacrylamide
BDDA: 1,4-butanediol diacrylate

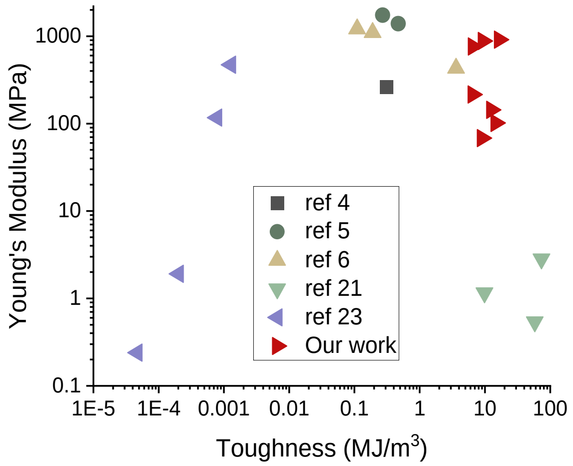

Supplementary Fig. 48 | Ashby plot of toughness vs. Young's modulus.

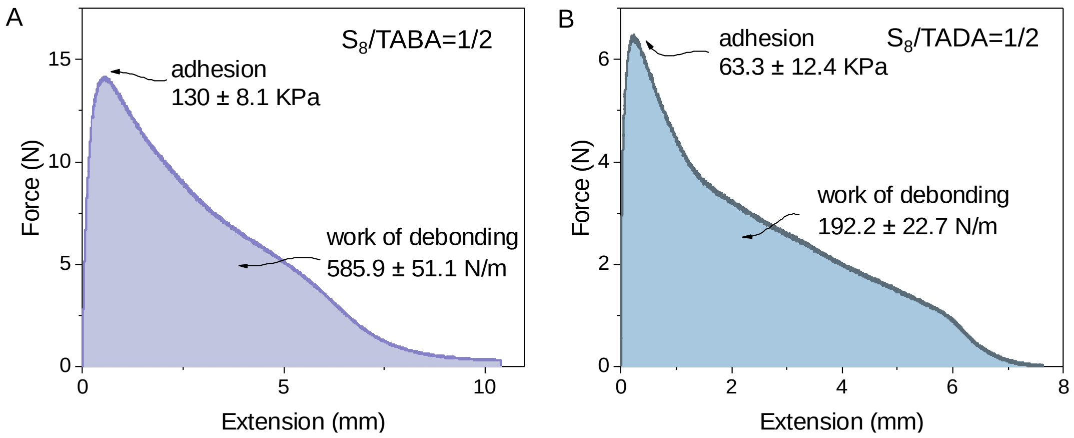

Supplementary Fig. 49 | Force-versus-extension curves for (A) poly(S_8/TABA=1/2) and (B) poly(S_8/TADA=1/2).

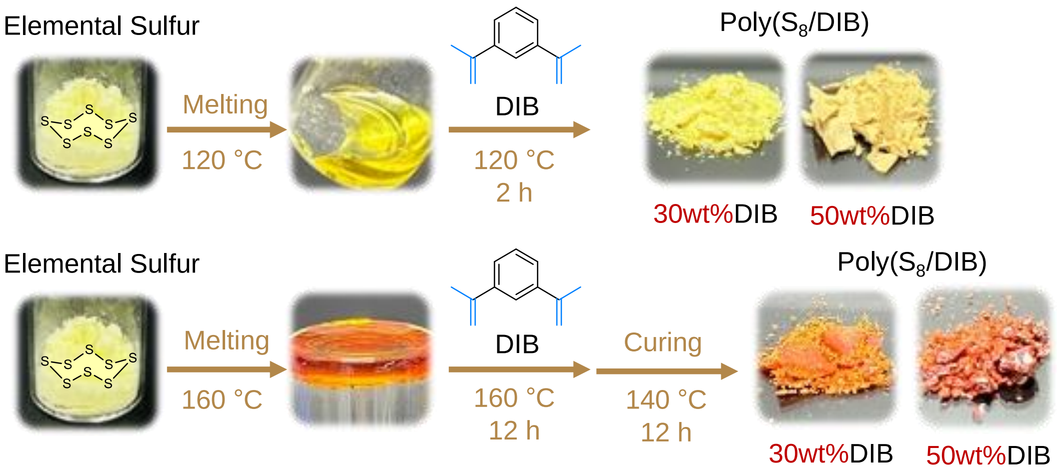

Supplementary Fig. 50 | Preparation procedure of sulfur rich polymers under different thermal condition.

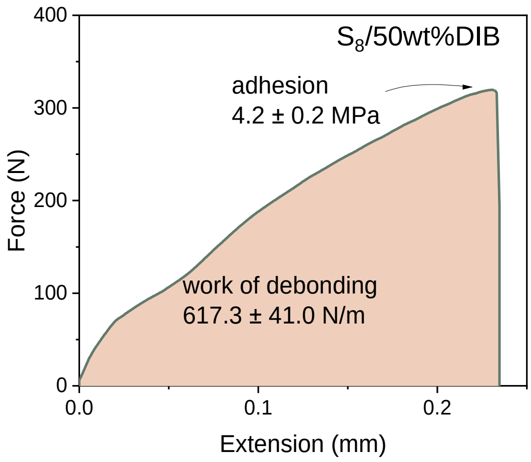

Supplementary Fig. 51 | Force-versus-extension curves for poly(S_8/50wt%DIB) prepared at 160 °C.

Supplementary Table 3 | A summary of the adhesion properties of the copolymer. The weight ratio of S_8 to TAA, TAH, TABA, and TADA is 1 to 2. Poly(S_8/50wt%DIB) prepared at 160 °C condition was chosen as the adhesive.

	Stainless Steel (MPa)	Aluminium (Mpa)	Glass (MPa)
S_8/TAA	6.40 ± 1.46	5.22 ± 0.21	5.21 ± 0.77
S_8/TAH	10.13 ± 0.60	10.91 ± 1.12	> 7.90 ± 0.96
S_8/TABA	0.13 ± 0.008	0.13 ± 0.01	0.06 ± 0.005
S_8/TADA	0.06 ± 0.01	0.07 ± 0.004	0.03 ± 0.01
S_8/50wt%DIB	4.26 ± 0.20	1.42 ± 0.51	2.21 ± 0.62

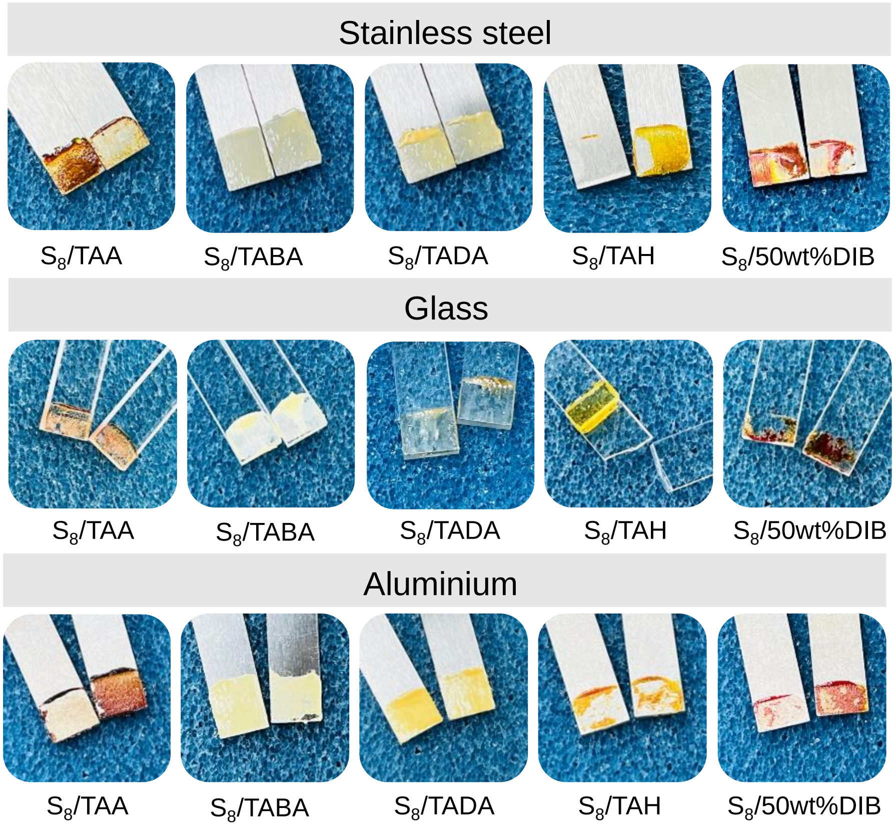

Supplementary Fig. 52 | Photographs of copolymers on various substrates after adhesion tests. The wight ratio of S_8 to TAA, TABA, TADA, and TAH is 1 to 1. The wight ratio of S_8 to DIB is 1 to 1.

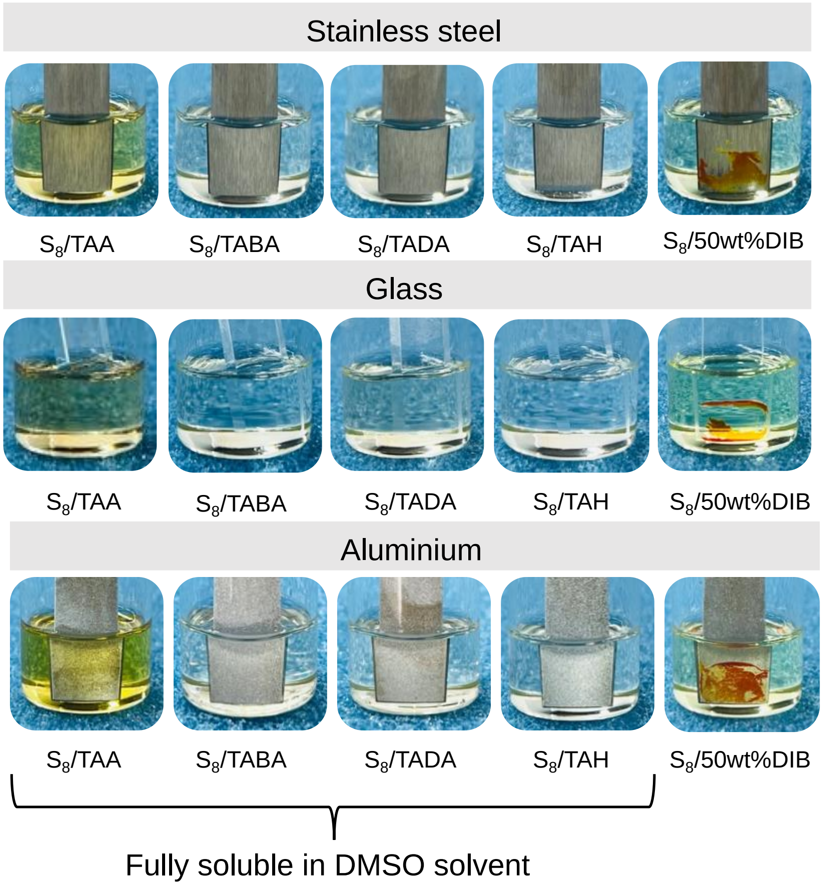

Supplementary Fig. 53 | Photographs of various adhesives after immersing in DMSO. Adhesives of poly(S_8/TAA), poly(S_8/TABA), poly(S_8/TADA), and poly(S_8/TAH) could be easily removed from the substrates after immersing them in DMSO for 30 min at room temperature. All substrates could be reused after cleaning. While poly(S_8/30wt%DIB) could not be fully removed from the substrates at room temperature even after 90 days. The wight ratio of S_8 to TAA, TABA, TADA, and TAH is 1 to 1. The wight ratio of S_8 to DIB is 1 to 1.

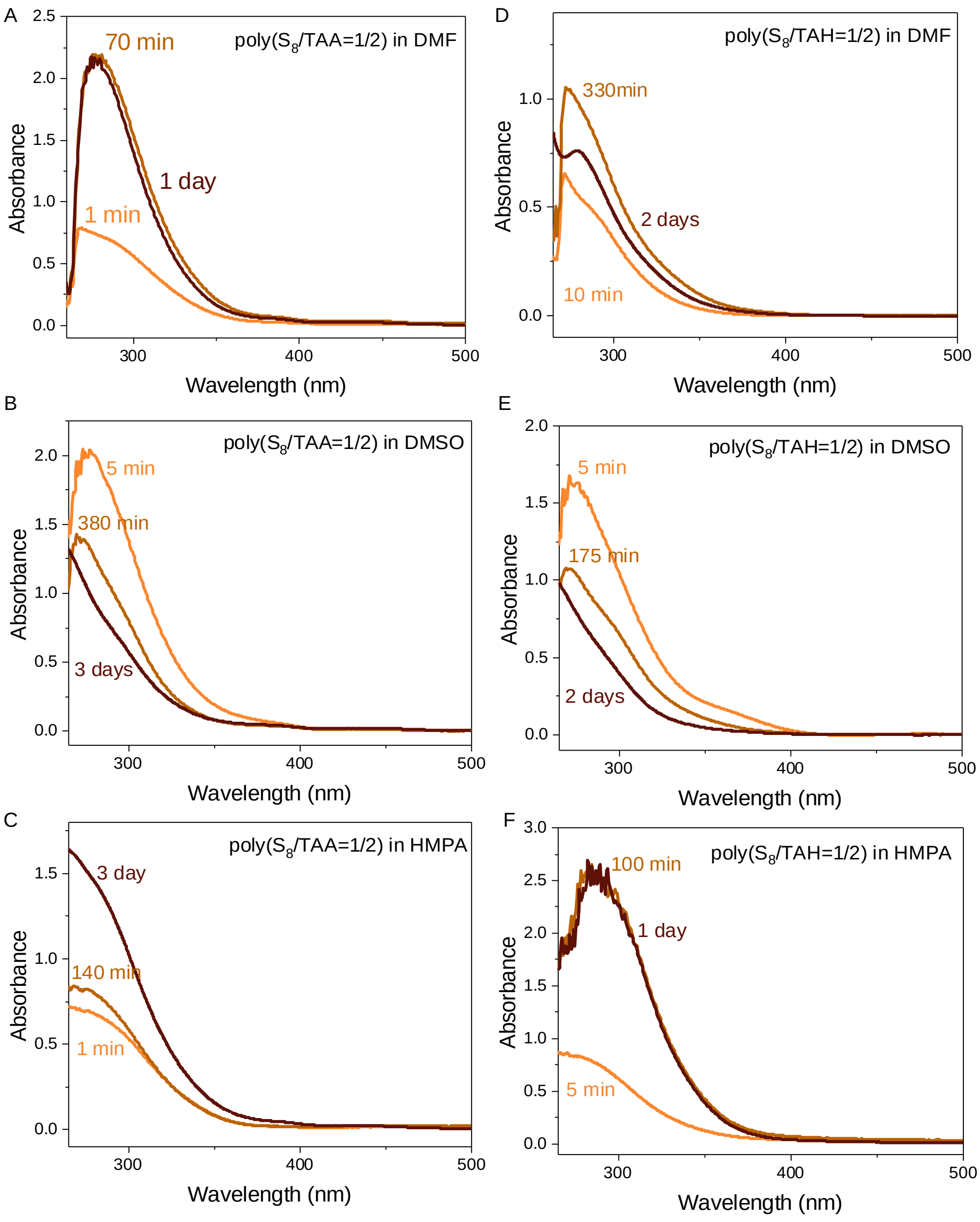

Supplementary Fig. 54 | UV-vis spectra analysis. The UV-vis spectra of poly(S_8/TAA=1/2) were measured in three different solvents: (A) DMF, (B) DMSO, and (C) hexamethylphosphoramide (HMPA). Similarly, the UV-vis spectra of poly(S_8/TAH=1/2) were measured in the same three solvents: (D) DMF, (E) DMSO, and (F) HMPA. All measurements were conducted at room temperature.

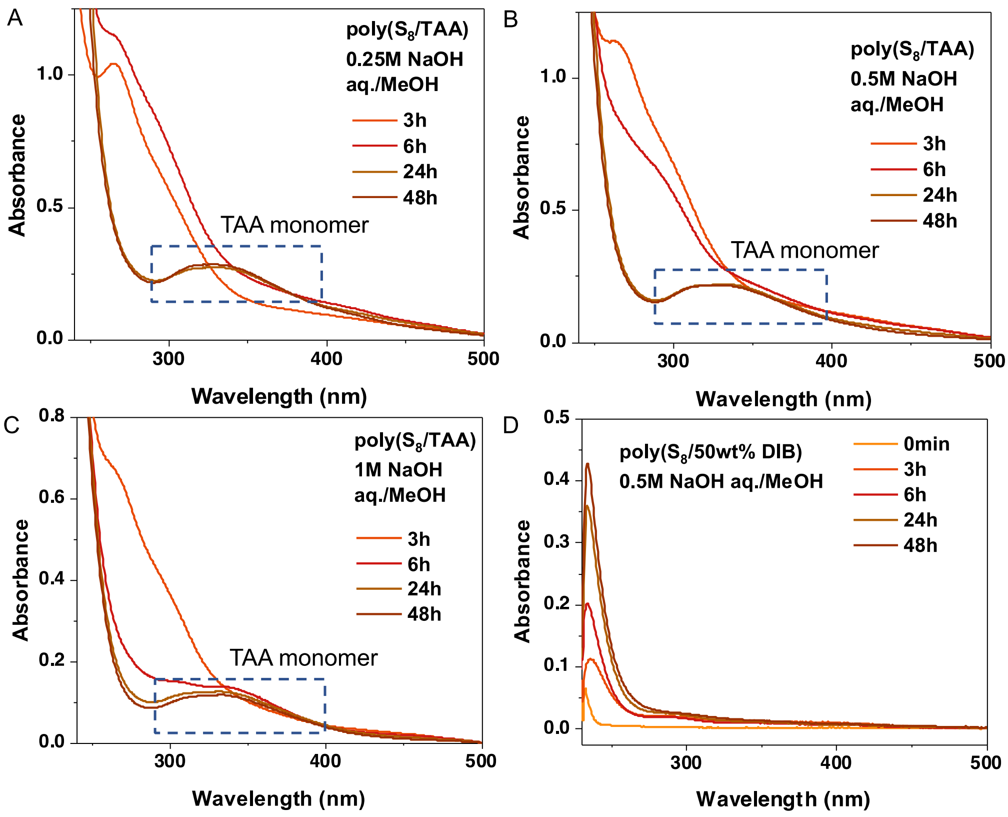

Supplementary Fig. 55 | UV-vis spectra analysis. (A-C) UV-vis spectra of degradable poly(S_8/TAA=1/2) were measured in three different base solutions, namely 0.25 M NaOH (A), 0.5 M NaOH (B), and 1 M NaOH (C). (D) UV-vis spectra of non-degradable poly(S_8/30wt%DIB) were measured in a base solution of 0.5 M NaOH. The measurements were carried out at room temperature in a solution composed of deionized water and methanol in a 1:1 volume ratio.

4. Supplementary Reference

1. Deng, Y. *et al.* Acylhydrazine-based reticular hydrogen bonds enable robust, tough, and dynamic supramolecular materials. *Sci. Adv.* **8**, eabk328, (2022).
2. Wang, B.-S. *et al.* Acid-catalyzed disulfide-mediated reversible polymerization for recyclable dynamic covalent materials. *Angew. Chem. Int. Ed.* **62**, e2022153 (2023).
3. Jia, J. H. *et al.* Photoinduced inverse vulcanization. *Nat. Chem.* **14**, 1249–1257 (2022).
4. Chung, W. J. *et al.* The use of elemental sulfur as an alternative feedstock for polymeric materials. *Nat. Chem.* **5**, 518–524 (2013).
5. Griebel, J. J. *et al.* Dynamic covalent polymers via inverse vulcanization of elemental sulfur for healable infrared optical materials. *ACS Macro Lett.* **4**, 862–866 (2015).
6. Kleine, T. S. *et al.* High refractive index copolymers with improved thermomechanical properties via the inverse vulcanization of sulfur and 1,3,5-triisopropenylbenzene. *ACS Macro Lett.* **5**, 1152–1156 (2016).
7. Lin, H.-K. & Liu, Y.-L. Reactive hybrid of polyhedral oligomeric silsesquioxane (POSS) and sulfur as a building block for self-healing materials. *Macromol. Rapid. Commun.* **38**, 201700051 (2017).
8. Diez, S., Hoefling, A., Theato, P. & Pauer, W. Mechanical and electrical properties of sulfur-containing polymeric materials prepared via inverse vulcanization. *Polymers*, **9**, 59 (2017).
9. Khawaja, S. Z., Vijay Kumar, S., Jena, K. K. & Alhassan, S. M. Flexible sulfur film from inverse vulcanization technique. *Mater. Lett.* **203**, 58–61 (2017).
10. Smith, J. A. *et al.* Crosslinker copolymerization for property control in inverse vulcanization. *Chem. Eur. J.* **25**, 10433–10440 (2019).
11. Wu, X. F. *et al.* Catalytic inverse vulcanization. *Nat. Commun.* **10**, 647 (2019).
12. Thiounn, T., Tennyson, A. G. & Smith, R. C. Durable, acid-resistant copolymers from industrial by-product sulfur and microbially-produced tyrosine. *RSC Adv.* **9**, 31460−31465 (2019).
13. Karunarathna, M. S., Lauer, M. K., Thiounn, T., Smith, R. C. & Tennyson, A. G. Valorisation of waste to yield recyclable composites of elemental sulfur and lignin. *J. Mater. Chem. A* **7**, 15683 (2019).
14. Park, S. *et al.* High strength, epoxy cross-linked high sulfur content polymers from one-step reactive compatibilization inverse vulcanization. *Chem. Sci.* **13**, 566 (2021).
15. Xin, Y. M., Peng, H., Xu, J. & Zhang, J. Y. Ultrauniform embedded liquid metal in sulfur polymers for recyclable, conductive, and self-healable materials. *Adv. Funct. Mater.* **29**, 1808989 (2019).
16. Kuwabara, J., Oi, K., Watanabe, M. M., Fukuda, T. & Kanbara, T. Algae-inspired, sulfur-based polymer with infrared transmission and elastic function. *ACS Appl. Polym. Mater.* **2**, 5173–5178 (2020).
17. Yan, P. *et al.* Inverse vulcanized polymers with shape memory, enhanced

mechanical properties, and vitrimer behavior. *Angew. Chem. Int. Ed.* **59**, 13371–13378 (2020).

18. Lauer, M. K., Tennyson, A. G. & Smith, R. C. Green synthesis of thermoplastic composites from a terpenoid-cellulose ester. *ACS Appl. Polym. Mater.* **2**, 3761–3765 (2020).
19. Smith, A. D., McMillen, C. D., Smith, R. C. & Tennyson, A. G. Copolymers by inverse vulcanization of sulfur with pure or technical-grade unsaturated fatty acids. *J. Polym. Sci.* **58**, 438−445 (2020).
20. Thiounn, T., Karunarathna, M. S., Slann, L. M., Lauer, M. K. & Smith, R. C. Sequential crosslinking for mechanical property development in high sulfur content composites. *J Polym Sci.* **58**, 2943–2950 (2020).
21. Kang, K. S. *et al.* Segmented polyurethanes and thermoplastic elastomers from elemental sulfur with enhanced thermomechanical properties and flame retardancy. *Angew.Chem. Int.Ed.* **60**, 22900–22907 (2021).
22. Yan, P. Y. *et al.* Mechanochemical synthesis of inverse vulcanized polymers. *Nat. Commun.* **13**, 4823 (2022).
23. Yan, P. *et al.* Stretchable and durable inverse vulcanized polymers with chemical and thermal recycling. *Chem. Mater.* **34**, 1167–1178 (2022).
24. Yang, H. J. *et al.* Anionic hybrid copolymerization of sulfur with acrylate: strategy for synthesis of high-performance sulfur-based polymers. *J. Am. Chem. Soc.* **145**, 14539–14547 (2023).
