# Supplementary material for: Converting inorganic sulfur into degradable thermoplastics and adhesives by copolymerization with cyclic disulfides
Source: Nat Commun. 2024 May 8;15:3855. doi: 10.1038/s41467-024-48097-4 (PMC11079033; doi:10.1038/s41467-024-48097-4)
Supplement: Supplementary file 3 — Description of Additional Supplementary Files [file 41467_2024_48097_MOESM3_ESM.pdf]

Description of Additional Supplementary Files

Supplementary Movie Legend:

Supplementary Movie 1: The present lap shear adhesive experiment was carried out on the glass substrate. The poly(sulfur)-based adhesive with sticky H-bond anchoring group exhibited strong adhesion performance making the glass stick broken during the lap shear test.
